# Exploring individual and organizational factors influencing cooperation in commons: a scoping review

**DOI:** 10.3389/fpsyg.2025.1465057

**Published:** 2025-06-03

**Authors:** Sabina Pedrazzini, Lilla M. Gurtner, Vincent Aggrey, Stephanie Moser

**Affiliations:** Centre for Development and Environment, University of Bern, Bern, Switzerland

**Keywords:** commons, cooperation, social dilemma, individual perspective, commons' organization, social identity, trust, social norms

## Abstract

Today, humanity faces multiple social and environmental crises that have arguably been caused by mainstream modes of economic organization. Against this background, commons represent a promising, viable alternative that enables people to self-organize to satisfy their needs in a more sustainable way. However, for commons to be successful, their members must cooperate. Despite the importance of cooperation for commoning processes, few studies in the scientific field of the commons have investigated cooperation using an individual-centered approach. To fill this knowledge gap, we conducted a scoping review to gather existing research about individual cooperation. We sought to identify factors that can impact cooperation in commons. We used a keyword search in three online databases to identify papers of interest. For inclusion, papers had to measure cooperation as an outcome variable, assess the impact of one or more factors on cooperation, use adult participants, and be written in English. The application of these criteria led to the inclusion of 135 papers. The included papers enabled us to identify nine factors influencing cooperation that could be divided into two categories. The first category includes individual factors, which depend on individuals' characteristics. These factors are: gender, social status, group identification, values and personality traits, and trust. The second category includes organizational factors, which concern the way individuals are organized as a group. These are: incentives, communication, social norms, and anonymity. We discuss these results vis-à-vis previous commons literature, showing that an individual perspective could significantly improve our understanding of how commons work. Moreover, we highlight the implications of the current review for future field research in commons.

## 1 Introduction

For several years now, humanity has faced multiple social and ecological crises, as highlighted by the latest report of the Intergovernmental Panel on Climate Change (Calvin et al., 2023) and the latest Global Sustainable Development Report (Independent Group of Scientists Appointed by the Secretary-General, 2023). It has been argued that these emergencies are a consequence of mainstream modes of social and economic organization, with policymakers' decisions relying on economic indicators such as Gross Domestic Product (GDP) without adequately considering social aspects or environmental limits (Raworth, 2012; Meadows et al., 2005). Raworth (2012, 2017) proposed a framework in which social and ecological goals are considered together. More specifically, she conceptualized a social foundation representing a boundary below which human wellbeing is not fully attained, and an environmental ceiling, beyond which ecological degradation takes hold. According to the concept, staying between these two boundaries would ensure “a safe and just space for humanity to thrive in” (Raworth, 2012, p. 4; see also Gupta et al., 2024).

In this context, commons^1^ represent a viable solution for social provisioning because they enable people to self-organize to directly address their needs in a more sustainable way (Bollier, 2014). By impacting both social and ecological aspects, commons could help humanity to move toward Raworth's “safe and just space”. Commons represent an economic organization beyond the classic private and public model (Ostrom, 1990). More specifically, commons can be defined as the integration of three key dimensions, equally important in the commons' definition (Bollier, 2014): first, a shared resource (Ostrom, 1990), or shared needs of a community (Euler, 2018). Second, a self-organized community of users (Ostrom, 1990), i.e., the commoners, that usually organizes in a local, egalitarian, and fair way (Gidwani and Baviskar, 2011). Third, the community's social practices, i.e., commoning, which enable creation of institutions to self-govern the commons (Euler, 2018; Schmelzer et al., 2022). Elinor Ostrom's seminal book “Governing the Commons” (Ostrom, 1990, p. 61–65) provides a classic example of commons from the Swiss alps, specifically from the village of Törbel. The account describes how peasants collectively own natural resources surrounding the village such as meadows and forests. Here, an association was created in 1483 to regulate the use of the communal lands by establishing specific rules regarding their access and exploitation. More specifically, this association set clear boundaries of the communally owned land and can decide if strangers who acquired land in the village can access the shared resources. Moreover, it established a simple rule to regulate the exploitation of the common lands, namely that one can send to the meadow only as many cows as they can feed during winter. Finally, the association also elects officials who must check that the rules are being respected by everybody, and who administrate fines if it is not the case. This set of practices ensures successful management of the communal land. Törbel's example highlights the importance of regulating individuals' behavior to avoid the overexploitation of the commons resource. Against this background, it is crucial to take a closer look at the commoners themselves, and at their internal organization, in order to gain a better understanding of successful, sustainable management of the commons.

Importantly, in order for a commons to be successful, its members must cooperate, i.e., they must incur a personal sacrifice for the greater good (Kollock, 1998). For instance, in the Swiss commons example, each of Töbler's peasants would derive greater personal gain by sending more cows to the meadow; at the same time, if they all sent more cows, the meadow would be overexploited, and this would have a negative impact for the whole community. The same logic applies to several higher-level societal problems, such as the overexploitation of natural resources, or the provision of public goods and services. In all these commons—be they at the community or at the global scale—every member faces a conflict between individual, short-term interests and collective, long-term ones. Acting for the commons' good requires a personal sacrifice, and the temptation to pursue individual interests is constantly present. Given (1) the role that commons could play in addressing social and ecological problems and (2) the fact that several global challenges have the same structure as commons, we need to understand the factors that enable cooperation, and how they can be fostered.

However, despite its importance, individual cooperation has scarcely been studied in the scientific field of commons, which mostly focuses on commoners' collective action and implementation of institutions to successfully manage the common resource (e.g., Ostrom, 1990; Poteete et al., 2010). Notably, however, researchers in other disciplines such as psychology and behavioral economics have extensively explored individual cooperative behaviors, for example through social dilemma experiments (i.e., standardized lab-based tasks to study under which circumstances individuals cooperate when personal and collective interests are at odds). In the following, we will present these literature strands, with particular attention to their specific focus.

One of the first papers on cooperation in commons was Hardin's “The tragedy of the commons” (Hardin, 1968). In this rather controversial article, echoing Malthusian fears of impending “overpopulation” and reflecting traditional economic conceptions of humans as self-interested, independent agents, the ecologist Hardin argued that users of commons are rational beings who will seek to maximize their personal interest by overexploiting the common resource. This will inevitably lead to the destruction of the resource, he maintained, unless an external institution regulates people's access, or the resource is privatized. Therefore, according to Hardin, people benefiting from a common resource cannot successfully manage it without external interventions.

In contrast to Hardin, several scholars have developed theories that focus on the ability of human beings to cooperate to manage a shared resource successfully. For instance, the Greed Efficiency Fairness hypothesis posits that individuals' greed-based impulse to pursue their own narrow interests is constrained by their desire to use the resource efficiently and to allocate it fairly (Wilke, 1991). Similarly, according to the Humanistic Rational Choice Theory, cooperation can be seen as the rational option when the institution in which people operate is accepted and perceived as legitimate, and when there is strong group cohesion (DeCaro et al., 2021). Moreover, later scholars have described Hardin's scenario as an oversimplification of the reality (e.g., Dietz et al., 2003) and argued that Hardin confused common properties with open access resources. In commons, there are rules that limit people's entry and use of the resource; these elements were not present in Hardin's argumentation (Dietz et al., 2002; Ostrom, 2008). Indeed, later research on commons has produced findings that refute the tragedy of the commons, demonstrating instead that people are capable of engaging in collective actions and managing shared resources in a sustainable way (Poteete et al., 2010). Among these researchers, Elinor Ostrom remains the most prominent to this day. Ostrom's seminal work (1990) highlighted several real-life commons that were successfully managed by the community of users without the intervention of an external authority. Based on her observations, she established seven design principles that were present in all the successful commons, plus an eighth one for more complex cases.^2^ These design principles have been widely applied to the analysis of other commons institutions in studies that have further confirmed their validity (e.g., Dietz et al., 2003; Tucker, 1999).

Ostrom's first principle stresses the centrality of boundaries: it must be clear what the shared resource is and who can benefit from it, otherwise “no one knows what is being managed or for whom” (Ostrom, 1990, p. 91). Second, the rules must be congruent with the local conditions, i.e., they must reflect the specific attributes of the particular resource. The third principle posits that individuals affected by the rules must be able to participate in modifying them. This ensures that the rules fit well to the local conditions and makes it possible to adapt them promptly when needed. The fourth and fifth principles concern monitoring and use of a graduated sanction system. Interestingly, these systems are in many cases successfully implemented by commoners themselves, avoiding the need for an external institution. Indeed, following the rules is the best individual strategy when others are also complying with them. In such a scenario, it is in everybody's self-interest to monitor other commoners. Similarly, the graduated sanction system can be easily self-regulated: fines are naturally small for the first infraction and increase only if the person persists on breaking the rules. In fact, since infractions are often due to extreme events, other commoners who must mete out punishment tend to be sympathetic because they could easily be in the same situation. The sixth principle posits that commoners must have access to low-cost conflict-resolution mechanisms, as disagreements between members are inevitable. Conflict-resolution arenas give the rule-breaker the possibility to explain their mistake, helping to ensure that compliance with rules does not diminish after every infraction. Finally, according to the seventh principle, external authorities should not challenge the commoners' right to self-organize and should recognize the legitimacy of commons institutions.

As indicated above, in the classical research on commons—based in real-life settings—commons are generally considered as a whole, and the perspective of individual commoners is largely absent from the discussion. Thus, improving our understanding of commoners' motivations to cooperate could expand and complement existing knowledge about commons management, including Ostrom's design principles. Meanwhile, disciplines such as psychology and behavioral economics have already studied cooperation in an individual-centered way. In these research strands, laboratory study of social dilemmas is very common and has enabled identification of several determinants of individual cooperation (e.g., Van Lange et al., 2013). A typical feature of these experiments (often also referred as “public good games” or “common resource experiments”) is that participants face a conflict between personal and collective interest. Each individual could obtain a greater personal payoff through non-cooperative, self-interested behavior as opposed to social, cooperative behavior, independent of what others do. At the same time, everyone's payoff would be higher if all cooperated rather than defected (Dawes, 1980). Social dilemma experiments are very useful for gaining insight into individual cooperation because they can be easily implemented in a controlled laboratory environment, enabling a standard experimental paradigm in which to explore the conditions under which people cooperate (Balliet et al., 2011b). Generally, the results of social dilemma experiments have demonstrated that people cooperate more than traditional economic models—which assume individuals' rationality and selfishness—would predict, and that their behavior can be influenced by psychological variables. For instance, the value of universalism, from Schwartz's model (1992) is associated with cooperation in studies where the participants must split an endowment between themselves and an interaction partner (Lönnqvist et al., 2013). Moreover, social dilemma experiments have shown that only about one third of participants behave in a purely selfish way—i.e., consistently making no contributions independent of how others behave. Around half of the participants in these experiments can be classified as “conditional cooperators”—i.e., they adapt their cooperation according to the amount contributed by others (Fischbacher et al., 2001; Thöni and Volk, 2018), even if the imperfect match of the contributions of others (“imperfect conditional cooperation”) leads to an overall decline in cooperation over time (Fischbacher and Gächter, 2010). Further, in an experimental study, Croson (2007) showed that the principle of reciprocity best explains individuals' cooperative behavior. In addition, some social dilemma experiments go in the same direction of the principles for a successful management of commons proposed by Ostrom (1990). For instance, Gürerk et al. (2006) demonstrated that when a sanction system is implemented, individual cooperation and individual payoffs are higher than when such a system is absent. Interestingly, in this study, almost all the participants preferred to switch to the sanction system over time.

Taken together, the above-mentioned theories and evidence support the notion that individuals can and do cooperate. However, the two literatures we explored have different focuses and methodologies. On the one hand, studies on commons focus on commoners' internal organization to manage the common resource. On the other hand, social dilemma experiments provide important insights into individual-level cooperation, but these experiments have rarely been implemented in real-world commons, i.e., their external validity has not been tested. For this reason, combining the rather descriptive, qualitative and institutional-centered approach of commons research with the quantitative, individual-centered perspective of social dilemma research could be beneficial to both commons and social dilemmas research strands. Combining these two literatures is relevant because commons and social dilemmas are deeply linked on a conceptual level, as they revolve around the same basic conflict between personal and collective interests (Dawes, 1980; Hardin, 1968). Indeed, Kopelman et al. (2002) used the term of “commons dilemma” to define situations in which a non-cooperative behavior seriously jeopardizes the future of a given shared resource. Commons have also been classified by Cumming (2018) as “take-some dilemmas”, a specific kind of social dilemma where people are tempted to increase their personal benefit by taking more than their fair share of a shared resource.

In our own literature review, we gathered existing research about individual cooperation in commons and in social dilemma experiments, to identify the state of the art regarding why and how people cooperate in commons. Giving their similarities, commons and social dilemmas have already been combined in some previous research. For instance, Hartl and Hofmann (2022) used a social dilemma framework to study real-life commons. However, the authors focused on one commons in particular, while the current review strives for more general results. Moreover, through a literature review, Kopelman et al. (2002) identified several individual and situational factors that affect cooperation in commons dilemmas. However, they limited their review to papers published in peer-reviewed psychology journals. For the current review, we did not exclude any discipline in advance, enabling us to provide a more complete overview of the factors impacting cooperation. Moreover, our scoping review encompasses more recent literature.

In conclusion, by synthesizing existing research on individual cooperation, our review strives to identify the most important factors shaping individual cooperative behaviors in commons. To our knowledge, this is the first review about this topic that considers both field-based and laboratory research. Thus, the present work can help: (a) to extend debate and theory regarding the design principles of commons by adding the perspective of individual cooperation; and (b) to implement experimental and quantitative studies in real-life commons, which, in turn, can enable important insights into cooperation beyond controlled laboratory settings. More concretely, our first goal with this research was to identify individual factors that shape cooperation in commons, thus showing that inclusion of an individual-centered perspective can aid understanding of cooperation in commons. Our second goal was to better understand which organizational factors (e.g., group strategies or self-chosen institutions) further shape individual cooperation. Simultaneously investigating the effects of institutions on individual behavior enables a fresh interpretation of findings in the commons literature. These concrete goals were translated into the following research questions:

1. Which individual factors affect people's cooperation in commons?
2. Which organizational factors affect individual cooperation in commons?

## 2 Method

We decided to perform a scoping review because this procedure enables collection of existing evidence on a specific topic in a broad way, thus allowing identification of key factors linked to the main concept (Munn et al., 2022). To conduct our scoping review, we followed the guidelines provided in the PRISMA extension for scoping reviews (Tricco et al., 2018). To be included in our review, papers needed to measure cooperation as a main outcome variable, and to assess the impact of one or more factors on it. Moreover, papers had to be written in English and use adult participants. Meta-analysis or reviews of several studies could also be included. Following the recommendations of Munn et al. (2022), to enable a broader account of relevant literature, the type of publication, the date, and the country where the research was conducted were not used as inclusion criteria. For the same reason, both quantitative and qualitative studies were included.

To identify articles for inclusion, we performed a Boolean search in Scopus, Web of Science, and ScienceDirect. As our goal was to include articles from different disciplines in our review, we decided not to use discipline-specific search engines such as PsychInfo. Our key search words were “commons”, “social dilemma”, “public good”, and “common resource”, paired with each of the following terms: “cooperation”, “conflicts”, “reciprocity”, and “trust”. Since our aim was to combine research about individual cooperation both in the laboratory and in the field, we wanted to keep a balance between commons-related and experiments-related keywords. For this reason, we selected a specific keyword for either setting (“commons” and “social dilemma”, respectively) as well as two keywords related to both (“public good” and “common resource”). To select the other keywords, we screened key literature to identify words that were frequently associated with “cooperation”.

The first author performed this research in August 2023, and again in November 2024, to include the relevant literature published after the first search. Moreover, in the final stages of the review process, we detected a problem with our original searches: specifically, the search engines we used treat the terms “good” and “goods” differently. For this reason, in February 2025, the first author re-performed the search of all the combinations of keywords containing “public goods”, using the plural form. This allowed us to identify additional papers that we missed in the previous rounds of search. More details about the procedure and the predefined search strategy can be found in Appendix A.

**Figure 1:**
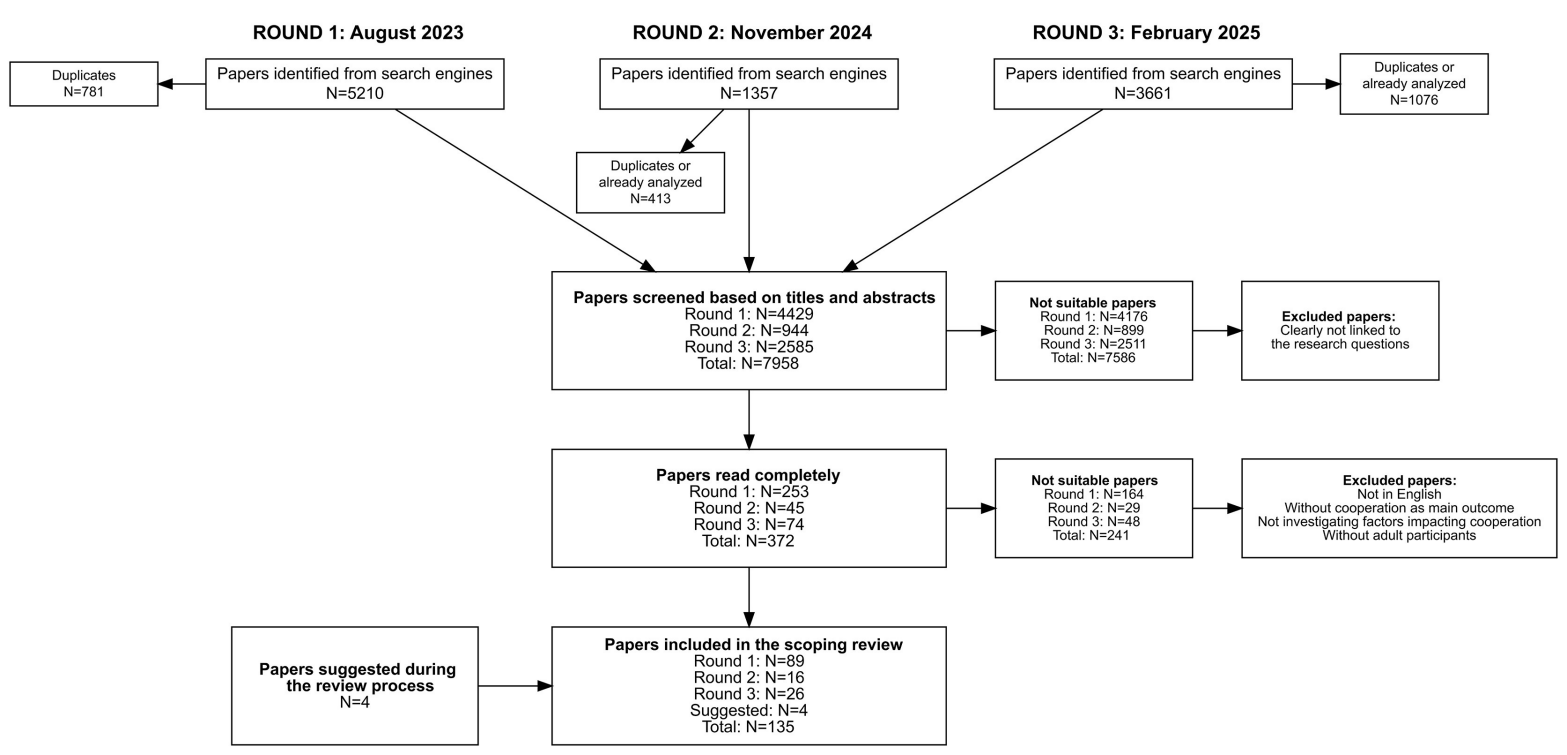
Overview of the text selection process. Source: authors' construct.

The selection process was conducted by the first author.^3^ Initially, the key word/phrases search identified 10,228 texts (all rounds confounded). Deleting duplicates reduced that number to 7,958. The first author then manually screened the titles and abstracts of the remaining papers, and excluded 7,586 of them because they did not focus on individual cooperation. This resulted in 372 texts to examine in total. Application of the inclusion criteria in the full-text screening resulted in the inclusion of 131 papers. Moreover, in the final stages of the review process we added four additional papers under the suggestion of the editor, which brought the final count to 135. Figure 1 summarizes this selection process and provides more details about the three different research rounds, while Appendix B provides more details about how the selected papers fulfill the inclusion criteria.

## 3 Results

In this section, we first describe the characteristics of the included papers. Second, we will present the identified factors that impact cooperation, either at the individual or organizational level. The first category refers to factors that depend on individual characteristics (such as personal values or trust), while the second refers to factors concerning how individuals are organized as a group (such as if incentives to cooperation are present or if individuals can communicate).

### 3.1 Description of papers

**Figure 2:**
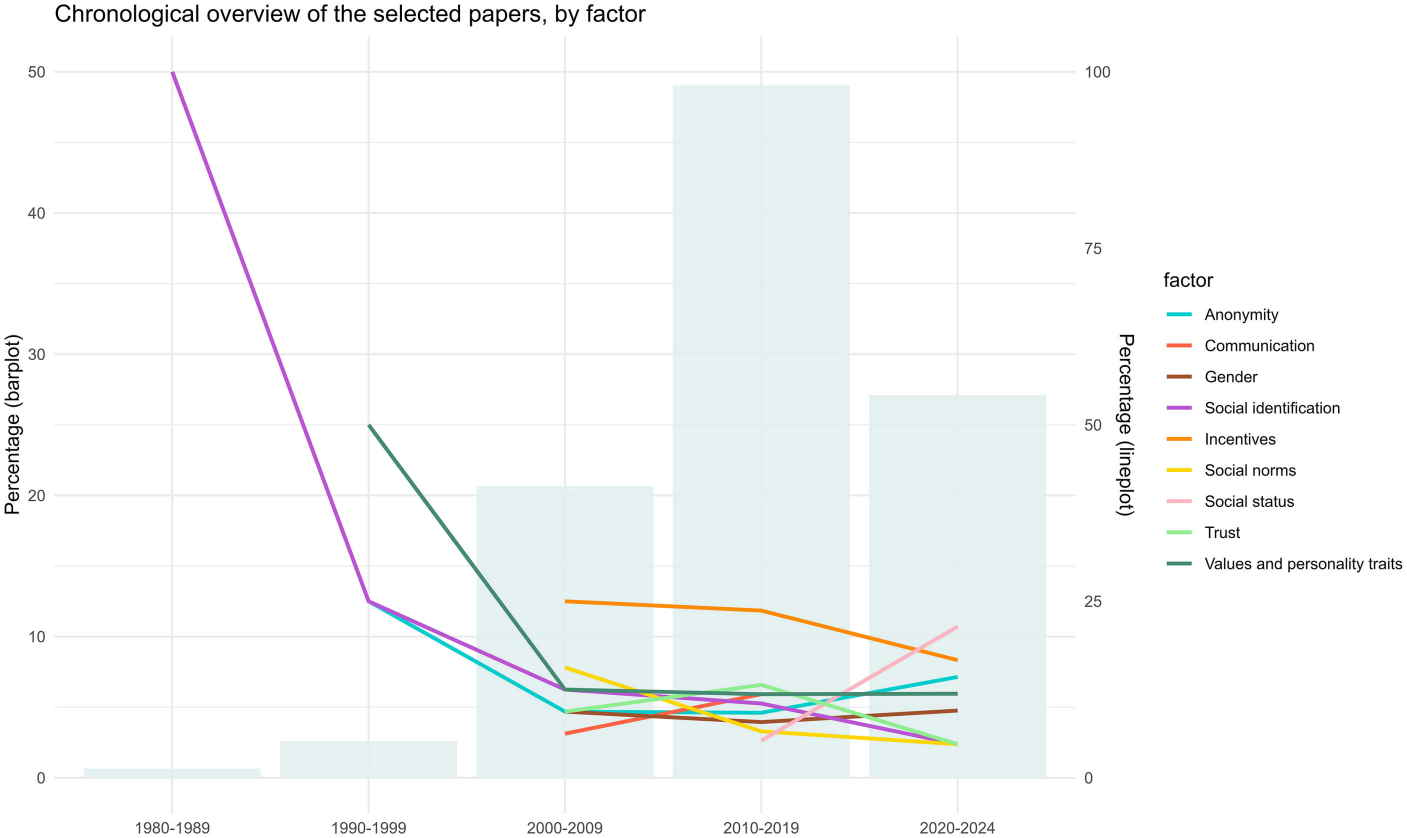
Chronological overview of the papers included in the current scoping review. Source: authors' construct, inspired from Bao et al. (2024). (1) The bar plot shows the distribution of the publication year of the papers included in our review. (2) The line plot shows the evolution of the publication trends over the year, by factor. The percentage is calculated using the total number of papers published in a decade.

Table 1 provides an overview of the 135 selected papers. Most were experimental studies (*N* = 102), followed by correlative studies (*N* = 16),^4^ meta-analyses (*N* = 9), and literature review (*N* = 7). The remaining paper was a qualitative study. Almost all were journal articles; the only exceptions were two book chapters. The years of publication ranged between 1984 and 2025, with a median of 2015. Figure 2 provides a chronological overview of the 135 papers included in the current review.

**Table 1:** Description of the selected papers.

**Setting**	**Methodology**					
	**Experimental**	**Correlational**	**Qualitative**	**Literature review**	**Meta-analysis**	**Total**
Lab-based social dilemmas	86	9	1	6	8	110
Lab-based commons	10	0	0	0	0	10
Real-life social dilemmas	2	5	0	0	1	8
Real-life commons	4	2	0	1	0	7
Total	102	16	1	7	9	135

#### 3.1.1 Setting and context

We identified two different dichotomous dimensions in the reviewed papers: setting (subdivided into lab-based vs. real-life) and context (subdivided into social dilemma vs. commons). There were papers in all four possible combinations, with the most represented category being that of papers focused on “lab-based social dilemma” (*N* = 110). In these studies, participants had to take cooperative decisions in a highly controlled laboratory setting. The second most represented category was “lab-based commons” (*N* = 10), i.e., studies utilizing a laboratory social dilemma experiment, but focused on a specific commons (e.g., Mosler, 1993). The category “real-life social dilemma” (*N* = 8) included papers investigating cooperation in different real-life situations with a social dilemma structure such as recycling behaviors (Rompf et al., 2017) or use of public transportation (Van Lange et al., 1998). Finally, the category “real-life commons” (*N* = 7) included studies of cooperation where participants were members of a commons in their everyday life.

#### 3.1.2 Journal

More than a half of the papers were published in psychology journals such as *Group Processes & Intergroup Relations* or *Journal of Environmental Psychology* (*N* = 40), in economics journals such as *Experimental Economics* (*N* = 30), or in interdisciplinary journals such as the *Journal of Economic Psychology* (*N* = 16). The remaining papers were published in journals concerning different topics, including biology (*N* = 6), mathematics and game theory (*N* = 4), sustainability (*N* = 4), and commons (*N* = 3).

#### 3.1.3 Country

Most of the studies were conducted in Europe (*N* = 58), especially in Germany (*N* = 22) and in the Netherlands (*N* = 11); followed by North America (*N* = 28), especially the US (*N* = 26); and lastly, Asia (*N* = 15), in particular China (*N* = 9). Moreover, nine studies were set in more than one country, and sixteen were meta-analyses or literature reviews. The nine remaining studies took place in New Zealand (*N* = 2), Australia (*N* = 2), Colombia, Paraguay, Namibia, Lebanon and Egypt, respectively.

#### 3.1.4 Operationalization of cooperation

The way cooperation was operationalized was closely linked with the study setting. Experiments conducted in a laboratory frequently observed participants' distribution of resources between themselves and a group account to quantify their level of cooperation. The most common method (*N* = 72) was to endow participants with an amount of a given resource (often real or fictive money) and observe what portion they invested in a public account from which the whole group could benefit. Otherwise, researchers observed what amount of the resource participants took for themselves from a collective pool (*N* = 11), or gave participants the choice between cooperation and defection (*N* = 22).^5^ In laboratory settings, connotated words like “cooperation” or “defection” were rarely used in the instructions given to participants, so as to avoid any influence on participants' responses.

In studies in real-life settings, the operationalization of cooperation was more often based on observed behaviors (*N* = 13). For instance, Rompf et al. (2017) used the amount of recycling behaviors as a proxy for cooperation, while in Van Lange et al. (1998) cooperation was represented by the habit of using public transportations to commute.

### 3.2 Individual factors

We defined as “individual factors” those elements that can vary from an individual to another in the same group. Our analysis identified five of them that impact cooperation: gender, social status, group identification, values and personality traits, and trust. In the following sections, we describe their main effects on cooperation and provide an overview of the moderators of each effect presented in the reviewed papers. A summary of the different individual factors is provided in Table 2 and further details about moderators are included in Appendix C.

**Table 2:** Summary of individual factors.

**Individual factors**	**Analyzed papers**	**General effect on cooperation**	**Moderators**
Gender	*N* = 13	Ambiguous	• Relationship with other participants • Intergroup comparison • Group composition in terms of gender • Underlying motives • External impact of cooperation
Social status	*N* = 13	Ambiguous	• Intra-class solidarity • Group composition
Group identification	*N* = 16	Strongly identified individuals cooperate more with the in-group than low-identified ones	• Intergroup competition • Super-ordinate identity • Other participants' individual contributions • Group size
Values and personality traits	*N* = 20	Pro-social individuals cooperate more than pro-self (Social Value Orientation) Pro-environmental values are positively correlated to cooperation Left-wingers are more cooperative than right-wingers Individual levels of justice sensitivity positively predict cooperation Collectivists act for the group's good, individualists act for their personal interest Individuals with higher self-control cooperate more Individuals scoring high in the Humility-Honesty dimension of the HEXACO model cooperate more than those scoring low Individuals scoring high in the Agreeableness dimension of the Big Five model cooperate more than those scoring low	• Incentives (SVO) • Uncertainty about the number of members (SVO) • Deliberation (SVO) • Group composition (SVO) • Other participants' behavior (pro-environmental values) • Type of cooperative interaction (political orientation) • Presence of a punishment system (justice sensitivity) • Presence of cooperative social norms (collectivism) • Effect of cooperation on personal and group outcomes (collectivism) • Amount of others' contributions (self-control) • Temptation to defect (Honesty-Humility)
Trust	*N* = 15	People with high levels of trust cooperate more than people with low levels	• Degree of conflict between personal and collective interests • Uncertainty about others' intentions • Anonymity • Group composition • Perceived costs and benefits of the cooperative behavior

The general effect on cooperation is reported as “Ambiguous” when the analyzed papers did not allow to draw a clear conclusion about how these factors affect cooperation. Otherwise, the main effect we identified is reported.

#### 3.2.1 Gender

In this category, we included studies that explicitly tested the difference between women's and men's cooperation or the effect of gender composition of the group on cooperation. Note that all the studies we mention in this section use a binary concept of gender. The results of these studies are contradictory: sometimes men cooperated more (Dorrough and Glöckner, 2019), while sometimes women did (Peshkovskaya et al., 2017). However, two meta-analyses reported a non-significant effect size (Balliet et al., 2011a; Spadaro et al., 2023). This indicates that gender differences in cooperation are very flexible and depend on the context. More specifically, Sell and Kuipers (2009) explained these contradictory results “by structural differences [of the experimental settings] and identities triggered by those differences” (p. 322).

Because of its variability, the effect of gender on cooperation is subject to several moderators. Specifically, the reviewed papers identified the following moderators: relationship with other participants, intergroup comparison, overall group composition, underlying motives, and impact of cooperation. First, men are more cooperative than women when they know other participants or can socialize before the cooperative interaction (Peshkovskaya et al., 2017). Moreover, men, but not women, increase their cooperation when they feel part of a team (Gomez-Ruiz and Sánchez-Expósito, 2020), or when they interact repeatedly with the same group (Balliet et al., 2011a). Second, when an intergroup comparison is salient, men display a stronger identification with their group and cooperate more than when such a comparison is not salient. This pattern is not observed in women (Van Vugt et al., 2007). Third, men-only groups are more cooperative than women-only groups (Colman et al., 2018; Peshkovskaya et al., 2019), whereas women cooperate more in mixed groups (Balliet et al., 2011a; Peshkovskaya et al., 2019). In general, mixed groups with a majority of women display the highest levels of cooperation (Spadaro et al., 2023), even if this effect of group composition can be moderated by other factors (e.g., Barrero-Amórtegui and Maldonado, 2021). Fourth, men are more susceptible to greed incentives than women—e.g., men cooperate less in situations where strong temptations to free ride are present (Simpson, 2003). On the other hand, women are more susceptible to the fear of being exploited and their cooperation is linked to their trust in other participants (Dorrough and Glöckner, 2019; Irwin et al., 2015). Finally, in a context where cooperation could potentially have a negative impact on external individuals, women, but not men, tend to reduce their levels of cooperation (Haucap et al., 2024).

Lastly, gender differences in cooperation could be partly explained by social status, with women representing the low-status group (Sell and Kuipers, 2009). For this reason, it is also important to gain insights into how social status impacts cooperation.

#### 3.2.2 Social status

In this review, we consider that a group is composed of individuals differing in social status if they do not have the same amount of resources before the cooperative interaction, if they do not receive the same benefits from this interaction, or if they have a different socio-economic background that is made salient in the study's context. The results of studies investigating the impact of social status on cooperation do not allow us to draw a clear conclusion about this effect. For instance, some findings suggest that low-status participants cooperate less in absolute terms, but more in proportional terms (i.e., they contribute a higher proportion of their endowments than high-status participants; Kingsley, 2016; Malthouse et al., 2023). However, other studies showed that wealthier people cooperated more (Peng and Fan, 2023; Van Lange et al., 2013).

As for gender, there are some moderating variables for the effect of social status on cooperation. More specifically, the analyzed papers highlighted the importance of intra-class solidarity and group composition. First, social status can create inter-class conflicts, which lead to lower overall cooperation levels, because people tend to cooperate more with members of the same social class (or ethnic group) than with members of other classes/groups (Aksoy, 2019; Waring and Bell, 2013). This is especially true for low-status groups, where solidarity can arise either in the form of discrimination toward high-status members (Camera et al., 2020), or in the form of fewer punishments for other low-status participants (Chen, 2022) and more punishments for high-status participants (Peng and Fan, 2023). Regarding the second moderator, overall group composition, studies disagree. On the one hand, some studies found that groups with an inequality of status between members displayed lower levels of cooperation than homogeneous groups (Banerjee, 2024; Aksoy, 2019). This can be explained by the fact that in heterogeneous groups, compared to homogeneous ones, the cooperative behavior is more strongly impacted by the expectations of others' cooperation (Drouvelis et al., 2021). On the other hand, another study showed that economic and socio-cultural heterogeneity are negatively correlated with in-group trust, but found no evidence of their effect on the integrity of a common-pool resource (Van Klingeren and De Graaf, 2021). Finally, increasing the endowments of low-status members to reduce inequalities is not enough to improve the group's levels of cooperation: in these groups, cooperation remains lower than in groups that were always homogeneous (Ramalingam and Stoddard, 2024a,b).

Taken together, these results suggest that the impact of social status on cooperation is complex. Moreover, similar to what Sell and Kuipers (2009) reported about the effects of gender and social status, Aksoy (2019) demonstrated that the lower level of cooperation between members of different social classes is mainly due to individuals' identification with their classes. This indicates that social identity and social status are deeply connected, and that it is difficult to separate their effects on cooperation.

#### 3.2.3 Social identification

Social identification refers to individuals' sense of belonging to a social group. In the context of this review, the relevant social group is that composed of individuals interacting in a cooperative situation. Social identification has a strong, positive effect on cooperation: the more individuals identify with a group, the more they cooperate with other members of the group (Van Vugt, 2009), for instance by exercising more voluntary activities for the collective good (Noonan et al., 2016). In fact, humans tend to have a highly developed sense of belonging and, when they strongly identify with a social group, they will be more concerned about the group's wellbeing and reputation (Van Vugt, 2009) and will value more group-related goals (Arora et al., 2016). Strong identification also leads to high levels of cooperation over time (Zhang, 2019). Finally, the strength of the effect of social identification on cooperation is further confirmed by two evidences: first, the effect is the same for individualist and collectivist people (Chen et al., 2007); second, group identification can be very easily induced. For example, Wit and Wilke (1992) grouped their participants before the experiment to determine their initial individual endowments, and this was enough to induce higher levels of cooperation compared to a condition where the endowments where determined separately for every participant.

Concerning moderators, the effect of social identification on cooperation depends on inter-group competition, super-ordinate identities, individual contributions of other participants, and group size. First, since people cooperate more with members of the same group than with members of other groups (Aksoy, 2019; Dorrough et al., 2015), intragroup cooperation can be enhanced by inducing a competition between different groups. In fact, when an intergroup comparison is present, cooperation is higher and decreases less over time (Böhm and Rockenbach, 2013). This positive effect can be explained by the fact that, in such a context, individual- and group-level interests are aligned (Puurtinen and Mappes, 2009). Moreover, a study showed that intergroup competition increased cooperation within the group, while individual, dispositional competitiveness had the opposite effect (Nockur and Pfattheicher, 2020). This demonstrated that the increase of cooperation via competition is due to group membership factors, and not to individual competitiveness. However, despite the observed beneficial effects of inducing intergroup competition, this could be harmful in real-life situations where resources are shared between several groups. In fact, there is a greater risk of overexploitation when an intergroup competition is present (Van Vugt, 2009). To maintain the positive effect of group identification while eliminating the risks associated with competition, it is useful to prime a superordinate identity (Van Vugt, 2009). This can be done by emphasizing the common characteristics of the different groups rather than their differences (Kramer and Brewer, 1984) or by inducing the feeling of a shared fate (Zhang, 2019). Cooperation can also depend on contributions of others, since individuals who strongly identify with their group can compensate, by cooperating more, for other in-group members' defection (Arora et al., 2016; Van Vugt, 2009). Finally, the group size can also moderate the impact of social identification on cooperation, since individuals' need to belong predicts cooperation in big, but not in small, groups (De Cremer and Leonardelli, 2003).

Lastly, social identification can also moderate the impact of other factors on the willingness to cooperate. For instance, monetary incentives are more effective in increasing cooperation in individuals with a low social identification (Van Vugt, 2009). Moreover, a previous group failure generally undermines cooperation, but this is not the case for individuals who strongly identify with the group (Jackson, 2011, 2012).

#### 3.2.4 Values and personality traits

Broadly speaking, personal values and personality traits are individual characteristics that play an important role in predicting cooperative behavior and can be measured using different psychological questionnaires (Hilbig et al., 2018). However, there are numerous values and personality traits, each requiring more specific definition. For this reason, we structure this section as follows: first, we focus on the particular effect of Social Value Orientation (SVO) on cooperation, which is the most investigated construct in the articles we analyzed. Second, we focus on additional values and, lastly, personality traits that also impact cooperation.

SVO makes it possible to distinguish between individuals who prioritize their own interests (“pro-self”) and those who prioritize group interests (“pro-social”; Balliet et al., 2009). The effect of SVO on cooperation has been widely demonstrated: in a meta-analysis, 81 of 82 papers reported that pro-social individuals cooperate significantly more than pro-self individuals (Balliet et al., 2009). The reviewed articles identified four moderators of SVO's main effect—namely incentives, uncertainty about the number of group members, possibility to deliberate, and group composition.

First, when economic incentives for cooperation are present, pro-self individuals attain a level of cooperation similar to pro-social individuals (Balliet et al., 2009; Emonds et al., 2011). More generally, pro-self individuals value the personal benefits of cooperative behavior more than pro-social individuals do (Van Lange et al., 1998). Second, being uncertain about the number of group members means not knowing how many people can exploit the shared resource. For this reason, if uncertainty is high, pro-social (but not pro-self) individuals increase their level of cooperation in order to avoid overexploiting the common resource (De Kwaadsteniet et al., 2008). Third, pro-self individuals increase their cooperation if they have the possibility to deliberate about the situation (Lu et al., 2019), while the effect of SVO on cooperation is even stronger if individuals are forced to think intuitively (i.e., they cannot focus exclusively on the cooperative task; Sun et al., 2023). However, Bilancini et al. (2022) found that the effect of SVO on cooperation is not affected by the cognitive load of participants, i.e., it does not change if participants are forced to take a quick decision. Finally, in groups with a heterogeneous composition in terms of SVO, cooperation decreases more strongly over time compared to homogeneous groups (Zhang et al., 2023).

As introduced above, SVO is not the only personal value that affects the cooperation of individuals. Indeed, in the articles we analyzed, we found that several other key values significantly impact cooperation, namely pro-environmental values, political orientation, justice sensitivity, allocentric values, and people's relative level of individualism–collectivism. More specifically, pro-environmental values (from the Schwartz Values Scale) positively predict cooperation, but only when participants confront non-cooperative interaction partners (Sussman et al., 2016). Moreover, political orientation also plays a role in predicting cooperation, with intention to vote for a left-wing party and accordance with left-wing policies being associated with more cooperation (Grünhage and Reuter, 2022). Another study using latent profile analyses to identify different profiles based on accordance with policies also found that participants labeled as “Progressive” (i.e., in accordance with left-wing policies) cooperated more than other participants, even if the differences with the other groups were not always statistically significant (Lönnqvist et al., 2025). A moderator of the effect of political orientation on cooperation is the type of cooperative interaction. Indeed, Fosgaard et al. (2019) found that individuals on the left side of the political spectrum cooperated more than individuals with right-leaning political preferences only in an experimental game in which participants were required to take resources from a common pool, and not when they must contribute to a public good. Cooperation is also positively correlated with people's individual level of justice sensitivity, that is, their readiness to perceive injustice. This effect is only observed in the absence of a punishment system (Schlösser et al., 2018). Additionally, allocentric (i.e., collectivistic) people cooperate more when a cooperative social norm is present (Chen et al., 2007). Finally, individualists tend to cooperate when it maximizes their personal payoff, whereas collectivists tend to act in ways that maximize the group's outcome (Probst et al., 1999). Interestingly, to some extent it is possible to modify people's level of collectivism or individualism by priming an independent or an interdependent “self-construal”, respectively (Liu and Li, 2009). The above-mentioned results allow to identify several moderators of the effects of personal values on cooperation, namely other participants' behavior (Sussman et al., 2016), the type of cooperative interaction (Fosgaard et al., 2019), the presence of a punishment system (Schlösser et al., 2018), the presence of cooperative social norms (Chen et al., 2007), and the effect of cooperation on personal or group outcomes (Probst et al., 1999).

Finally, personality traits can also influence individuals' cooperation. The articles reviewed highlighted the effects of self-control, agreeableness, and humility-honesty. More specifically, individuals with higher levels of self-control cooperate more in comparison with individuals scoring lower on this trait. Moreover, this effect becomes stronger with the increasing of others' contributions (Kocher et al., 2017). The Agreeableness dimension of the Big Five model also positively predicts cooperation (Volk et al., 2011). Additionally, the Honesty-Humility trait from the HEXACO model of personality plays an important role in cooperation: individuals scoring high in this trait display more cooperation and show more hesitation before making a non-cooperative choice (Kieslich and Hilbig, 2014). This effect is moderated by the game structure, since the Honesty-Humility trait successfully predicts cooperation only in situations where a strong temptation to defect is present (Hilbig et al., 2018). These results allowed us to identify the following moderators of the effect of personal values on cooperation: the amount of others' contributions (Kocher et al., 2017) and the presence of a temptation to defect (Hilbig et al., 2018).

#### 3.2.5 Trust and beliefs about others' contributions

In the present article, we define trust as the “expectations of others' benevolent motives in situations that involve a conflict between self and collective interests” (Balliet and Van Lange, 2013a, p. 1). There are two categories of trust: first, “dispositional trust” is an individual, general trait and can be assessed by means of a personality scale. Second, “state trust” is context-dependent and represents one's expectations regarding a partner's behavior in a specific situation (Balliet and Van Lange, 2013a). The two are positively correlated (Lübke, 2021) and both have a positive effect on cooperation (Balliet and Van Lange, 2013a; Van Lange et al., 2013). Several studies have confirmed this relationship: high levels of trust are associated with greater cooperation (Kocher et al., 2015); at the same time, individuals who cooperate more display higher levels of trust in subsequent interactions (Chaudhuri et al., 2002). Similarly, the cooperation of individuals is associated with their beliefs about the contributions of others (Smith, 2013; Bechtel and Scheve, 2017; Oyediran et al., 2018): specifically, they tend to contribute slightly less than what they think others did (Irlenbusch et al., 2019). Finally, the more individuals believe that other people are helpful and the more they trust strangers, the higher their levels of cooperation (Gächter et al., 2004). The enhancing effect of trust on cooperation has also been demonstrated in studies in real-life settings (e.g., Franzen et al., 2019; Sturm et al., 2019).

In the studies reviewed, we identified four moderators of the effect of trust: conflict of interest, uncertainty about others' intentions, anonymity, and group composition. More specifically, trust matters more in situations involving a large—rather than small—conflict between personal and collective interests (Balliet and Van Lange, 2013a) as well as when people are uncertain about others' intentions (Van Lange et al., 2013). Further, individuals with a low degree of trust increase their cooperative contributions when their actions are accountable, as opposed to anonymous. This is not the case for “high trusters”, who display high levels of cooperation no matter the level of transparency (De Cremer et al., 2001). In addition, trust has a larger impact on cooperative behaviors in heterogeneous, rather than homogeneous, groups (Drouvelis et al., 2021).

Finally, people's beliefs in others' cooperation can be modified by providing feedback about previous cooperation (Irlenbusch et al., 2019). People can also display varying levels of trust in the “reliability, effectiveness, and legitimacy of public institutions” (Rompf et al., 2017, p. 2), and this institutional trust also positively correlates with cooperation.

### 3.3 Organizational factors

We defined as “organizational factors” those aspects linked to the group's organization. These factors can be further divided into two categories: self-chosen institutions and characteristics of the interactive situation. The first category includes rules and mechanisms that the group decides to implement in order to regulate internal organization and promote individual cooperation. From the papers reviewed, we identified two factors in this category: incentives and social norms. The second category of organizational factors refers to characteristics of the interactive situation that can influence individual cooperation. In this category, the factors we identified were communication and anonymity. In the following sections, we detail the main effects of each organizational factor and provide an overview of possible moderators. A summary of the main effects is provided in Table 3; additional details about the moderators can be found in Appendix C.

**Table 3:** Summary of organizational factors.

**Organizational factor**	**Analyzed papers**	**General effect on cooperation**	**Moderators**
Incentives	*N* = 33	Punishment and reward systems have a positive effect on cooperation	• Cost of implementation • Type of interaction • Other participants' characteristics and group homogeneity • Organization • Accuracy of information about others' contributions • Cultural context • Type of incentives
Social norms	*N* = 12	Cooperative social norms increase cooperation	• Anonymity • Content of the descriptive norm
Communication	*N* = 16	Having the possibility to communicate with other members increases cooperation	• Group size • Type of communication • Content • Timing • Context
Anonymity	*N* = 17	Individuals cooperate more in non-anonymous situations than in anonymous ones	• Personal costs and benefits • Individual level of image concern • Trust

#### 3.3.1 Incentives

In the selected articles, incentives refer to punishments or rewards that participants receive according to how they behave in the cooperative interactions. Two meta-analyses showed that introducing a punishment or reward system can increase cooperation (Balliet et al., 2011b; Jin et al., 2024). Both systems have a positive effect on cooperation, but it is not clear which one works better: some studies found that punishments are more efficient than rewards (e.g., Milinski and Rockenbach, 2012), while others came to the opposite conclusion (e.g., Chen, 2022)—also because rewards lead to higher group payoffs than punishments (Rand et al., 2009). In general, punishments have been studied more than rewards (Van Dijk et al., 2015), possibly because Fehr and Gächter's (2000) seminal experiment provided an efficient experimental setup to investigate the effect of punishment on cooperation. In this experiment, the researchers showed that when participants can punish others, cooperation increases and stays high over time. The success of punishment systems in increasing cooperation could be explained by how they change participants' perception of the situation. Indeed, Nockur et al. (2021) found that cooperative interactions are perceived as fairer under any form of punishment system, compared to conditions without punishment.

Several factors moderate the main effect of incentives^6^ on cooperation, namely the cost of implementation, the type of interaction, other participants' characteristics and group composition, the organization, the accuracy of information about others' contribution, and the cultural context. More specifically, incentives are more effective when their implementation is costly rather than free and when they occur in iterated, rather than single, interactions (Balliet et al., 2011b). Indeed, in experiments where participants interact repeatedly and have the opportunity to punish each other, cooperation converges toward the maximal level in the last rounds (Fehr and Gächter, 2000). Punishments also work better in groups where all members have the same income (Chen, 2022; Kingsley, 2016) and when participants are friends (Balliet et al., 2011b; Jin et al., 2024). At the same time, punishment tends to be lower in mixed groups—i.e., groups composed of participants with different socio-economic backgrounds—and this has a negative effect on cooperation (Drouvelis et al., 2021). In terms of organization, the punishment network—i.e., who can punish who—influences the effectiveness of punishment (Peng and Fan, 2023). More specifically, the most effective configuration is when (solely) those who cooperate have the power to punish defectors (Xiao and Kunreuther, 2016). In addition, the link between behavior and outcome must be clear (Xiao and Kunreuther, 2016), the probability of receiving the punishment after defecting must be high (Almeida, 2023), and the punishments must be significant (Egas and Riedl, 2005). Interestingly, these conditions seem to appear spontaneously during the cooperative interaction: Fehr and Gächter (2000) demonstrated that the lower the cooperation of a subject, the heavier the punishment they receive. Moreover, in the long run, graduated punishments—i.e., small for the first infraction and gradually increasing—are more efficient than non-graduated punishments (Van Klingeren and Buskens, 2024). Also, to be more effective, punishments should be applied with care (Rockenbach and Wolff, 2019). Concerning the accuracy of information about others' behavior, two studies found that punishment is more effective in improving cooperation when participants are informed about others' exact contributions. Indeed, in conditions where the information about others' contributions was not exact, participants tended to punish more, but this did not increase cooperation (Grechenig et al., 2010). This can be explained by the fact that participants who cooperated but were punished anyway due to inaccurate information are less likely to cooperate in the following interactions (Ambrus and Greiner, 2012). Finally, concerning the general cultural context, the effectiveness of punishment depends on the overall societal level of trust (Balliet and Van Lange, 2013b) and on the cultural norm of civic cooperation (Herrmann et al., 2008). In particular, punishment is more effective in cultures with higher levels of trust, and with a strong cultural norm of civic cooperation, which is negatively correlated with the use of “antisocial punishment” (i.e., punishment of cooperators by defectors).

Incentives can also take a social form: expecting honor or shame (after cooperating or defecting, respectively), expressing disapproval toward defectors, and excluding defectors from interactions can all enhance cooperation (Jacquet et al., 2011; Maier-Rigaud et al., 2010; Nelissen and Mulder, 2013). Moreover, if there is the possibility of being excluded, cooperation does not decrease toward the end of the interaction (Cinyabuguma et al., 2005). However, the reviewed studies provided contradictory evidence about which form of incentives (social or economic) is more effective. On the one hand, in groups where economic incentives are present and then removed, cooperation is lower than in groups that never had incentives to begin with Chen et al. (2009); Mulder et al. (2006). The removal of social incentives does not have the same detrimental effect on cooperation (Nelissen and Mulder, 2013), thus suggesting that social incentives could be more effective. On the other hand, the effect of social sanctions diminishes over time, in contrast to economic incentives (Noussair et al., 2024).

Finally, despite initially enhancing cooperation, incentives can have negative side effects: they reduce participants' intrinsic motivation to cooperate (Van Dijk et al., 2015) by framing the situation as an economic rather than an ethical dilemma (Van Vugt, 2009). Indeed, when a punishment system is implemented, individual characteristics impacting cooperation (such as values or personality traits) have a weaker effect than when such a system is not present (Schlösser et al., 2018; Hilbig et al., 2012). Incentives also undermine participants' trust in other members' internal motivation to cooperate (Irwin et al., 2014). Moreover, a costly punishment system could entail a second-order social dilemma because those who cooperate may still be incentivized to save by not investing their resources in meting out punishment (Milinski and Rockenbach, 2012). Also, in such a system, the total group earnings are lower (Wu et al., 2016)—especially in the first rounds of the interaction (Fehr and Gächter, 2000). Moreover, when all participants can punish each other, there is the risk of antisocial punishment (defectors punishing cooperators), which undermines cooperation (Herrmann et al., 2008). A democratic punishment system, in which a fine is given only when a majority agrees, could solve these problems (Pfattheicher et al., 2018).

#### 3.3.2 Social norms

Social norms refer to informal norms that can regulate social life by providing guidance on how to behave. Social norms can be divided into “injunctive norms” (what others approve of) and “descriptive norms” (how others actually behave), and both impact cooperative behaviors (Thøgersen, 2008). Social norms are especially important when there is uncertainty about others' behavior, as in cooperative situations (Von Borgstede et al., 2018). Social norms shape cooperation because, when taking a decision, people ask themselves: “What does a person like me do in a situation like this?” (Arora et al., 2012). If they define the situation as a cooperative task and strongly identify with the group, the group's norms will be more accessible and they will have a stronger impact on behavior (Van Lange et al., 2013). Moreover, people have a general tendency to imitate what others do, even if others' actions do not have an impact on their personal gains (Bardsley and Sausgruber, 2005).

We identified two moderators: anonymity and content of the norms. First, if behaviors are observable, people comply more with the injunctive social norms of cooperation and, therefore, cooperate more (Rege and Telle, 2004). Second, an experiment manipulating the descriptive social norm demonstrated that if the rest of the group cooperates, participants adapt and take their fair share of the shared resource; at the same time, if the descriptive norm is to over-harvest the shared resource, participants try to communicate the cooperative norm by harvesting less than their fair share (Lavallee et al., 2024). The papers we reviewed also highlighted several ways to modify social norms. First, in a context where the descriptive social norm is to defect, cooperation can be encouraged by inducing a feeling of pride in the few cooperators, or by appealing to the defectors' social values (Hassan et al., 2023). More generally, appealing to participants' goodwill can effectively increase cooperation (Chaudhuri and Paichayontvijit, 2017). Social norms can also be directly modified by participants' behavior. For instance, the presence of consistent cooperators (i.e., people who always cooperate, independently of others' actions) creates a more cooperative descriptive social norm and thus increases cooperation (Weber and Murnighan, 2008). Moreover, participants can also shape cooperative social norms via communication (Bicchieri, 2002). Finally, providing feedback about others' contributions is also an effective way to make the descriptive norm salient: in an experimental setting, Irlenbusch et al. (2019) showed that the higher a contribution (of a single individual) communicated to the other participants, the higher the cooperation in the following round. This positive effect of feedback on cooperation is mediated by participants' trust in others.

#### 3.3.3 Communication

In the papers included in our review, communication is operationalized by allowing participants to interact (in-person or via written messages) before, during, or after the cooperative interaction. Opportunities for communication between members have a general positive effect on cooperation (e.g., Balliet, 2010; Jin et al., 2024) because they make it possible to shape a cooperative social norm (Bicchieri, 2002; Janssen et al., 2014) and to reach an agreement about the best strategy for the group (Hopthrow and Hulbert, 2005). Moreover, communication has a positive effect on cooperation even if the participants exchange in an anonymous form (Baum et al., 2012).

The papers analyzed identified group size, type of communication, content, timing, and context as moderating effects. First, communication between members has a stronger impact in large groups rather than small ones, and second, oral communication is more powerful than written messages (Balliet, 2010). Moreover, if communicating requires a personal financial cost, its effect is weaker than if communication is free (Janssen et al., 2014). Third, concerning the content, to be effective communication must enable participants to become aware of the problem faced by the group, identify different solutions, and agree on the solution to adopt (Koessler et al., 2021a). Communication also has a stronger effect when it is used to foster a common social identity, promote cooperative social norms, and ensure that every member publicly commits (Adams et al., 2022). Moreover, even communication about topics that are unrelated to the cooperative task improve cooperation, even if task-related communication is more effective (Hoenow and Pourviseh, 2024). Concerning the timing, communicating before or during the cooperative interaction has a positive impact on cooperation (Balliet, 2010), whereas knowing that it will be possible to communicate after the interaction does not enhance cooperation (Kumakawa, 2013) and can even have a negative effect (Torsvik et al., 2011). Finally, in a real-life context where participants already know each other, the positive effect of communication is weaker, since cooperation is high even in absence of communication (Ghate et al., 2013).

Finally, one particular form of communication is gossip, i.e., the spread of reputational information about another person (Feinberg et al., 2014). Gossip tends to increase cooperation by providing information about the past behavior of potential partners, which helps to select the most cooperative members in the following interaction. Moreover, the prospect of gossip motivates people to cooperate more to avoid being the target of such gossip (Feinberg et al., 2014). Gossip can also have the pro-social function of protecting others from potential exploitation (Feinberg et al., 2012). Providing correct information while gossiping also helps to build a positive reputation (Giardini et al., 2021) and reduces negative feelings on the gossip actor (Feinberg et al., 2012). Finally, gossip improves not only cooperation, but also the total group's payoff (Wu et al., 2016).

#### 3.3.4 Anonymity

In the context of this review, a situation is considered anonymous when group members cannot monitor or trace the behavior of each other. In general, cooperation is significantly higher in non-anonymous situations than in anonymous ones (Wang et al., 2017; Hill and Gurven, 2004). This can be explained by two mechanisms: first, if relevant behaviors are visible, there are more social incentives to cooperate because people worry about the approval of others (Rege and Telle, 2004). This reputational effect is extremely powerful: a real-world study showed that making public the names of the participants in an intervention that would benefit the whole community was more effective in convincing people to participate than offering the participants a moderate economic incentive (Yoeli et al., 2013). Second, individuals are more willing to cooperate when they have more information about their interaction partner (Ma et al., 2024).

The articles reviewed highlighted three moderators to the effect of anonymity on cooperation, namely personal costs and benefits, personal level of image concern, and trust. First, the positive effect of non-anonymity is stronger in situations where cooperation bears a cost without offering personal gain (Butz and Harbring, 2021; Van Vugt and Hardy, 2010); when behaviors are visible, cooperation can serve as a self-presentation strategy to be perceived as pro-social. Second, making a situation non-anonymous has a stronger positive effect on cooperation among individuals possessing higher levels of image concern (i.e., concern about what others may think of them; Christens et al., 2019). Third, anonymity has a stronger negative impact on the cooperative behavior of individuals who possess low levels of trust (De Cremer et al., 2001).

Finally, we identified two ways to reduce anonymity in cooperative interactions. First, it is useful to make people promise to cooperate in a public way. Such a commitment significantly increases cooperation rates, even when mechanisms to enforce subsequent cooperation are lacking (Mosler, 1993; Przepiorka and Diekmann, 2020). Moreover, even a compulsory commitment has a beneficial effect on overall rates of cooperation (Koessler et al., 2021b). Second, some results suggest that reducing group sizes could also reduce anonymity, as larger groups lead to de-individuation that makes it harder to identify defectors (Romano et al., 2016). However, the link between group size and cooperation has not been systematically confirmed: on the one hand, Jiang et al. (2021) demonstrated that cooperation levels are higher in smaller groups. On the other, in a recent meta-analysis, the size of the group was not associated with cooperation (Jin et al., 2024). Moreover, Weimann et al. (2019) demonstrated that large groups can also cooperate successfully; here, the effect of the group size on cooperation depends on the personal benefits that can be derived from cooperative behavior. More specifically, in large groups, the advantages of cooperation must be evident for individuals to be willing to cooperate.

### 3.4 Connections between factors

**Figure 3:**
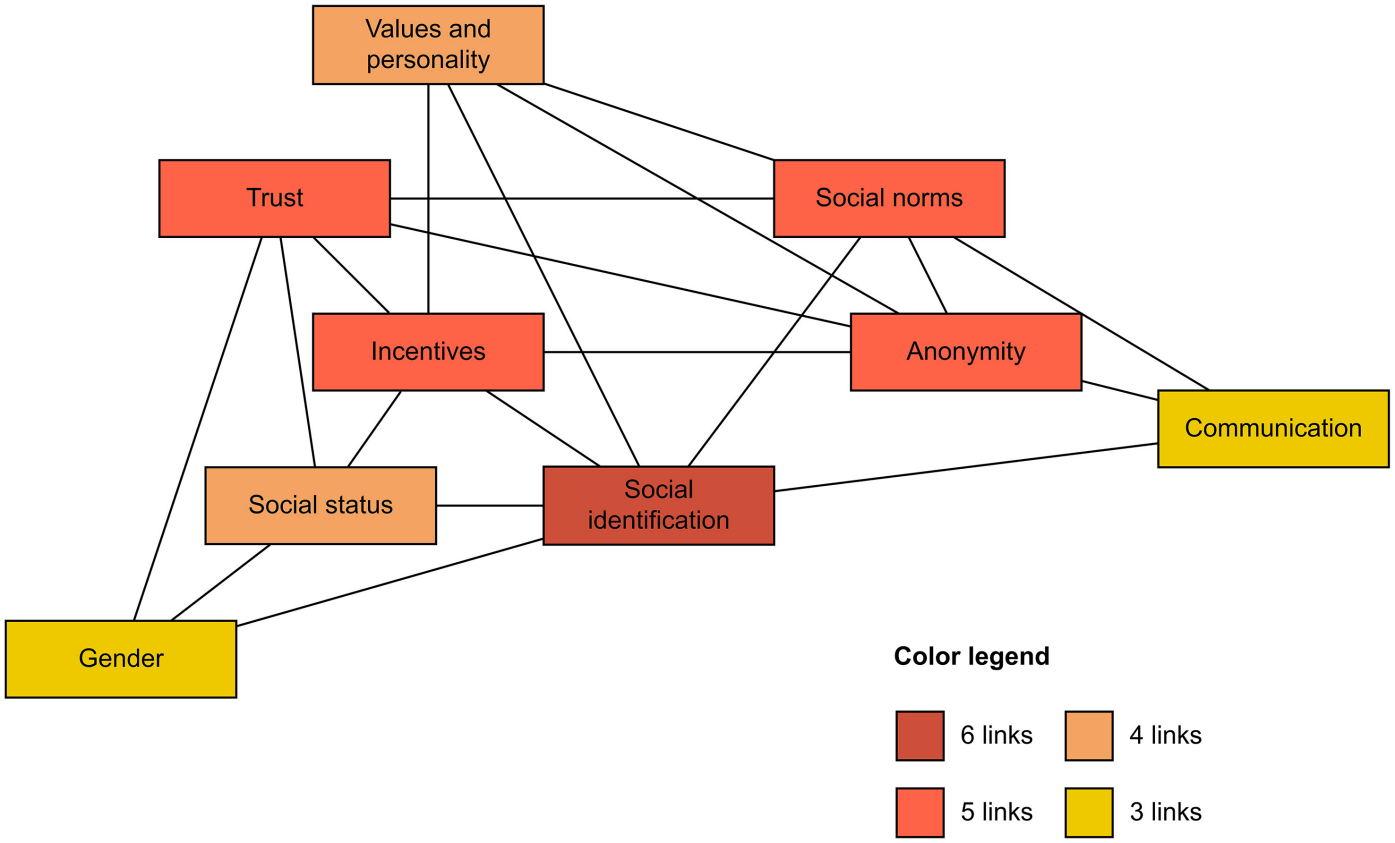
Connections between the nine factors identified in the current scoping review. Source: authors' construct.

Our analysis also revealed several connections between the nine factors we identified. We define two factors as “connected” if one moderates or mediates the effect of the other on cooperation. These connections are already mentioned in the previous sections, but not comprehensively. The aim of this section is to enumerate these links and provide an overview of how the nine factors we identified relate to each other. Figure 3 offers a summary of existing connections between the factors.

Above all, social identification is the most interconnected factor, displaying links with social status, social norms, incentives, personality traits, communication, and gender. Indeed, social identification partly explains the effect of social status on cooperation (Aksoy, 2019) and makes group norms more accessible and influential with regard to individual behavior (Van Lange et al., 2013). Social identification also moderates the impact of incentives and of individualism on cooperation: individuals who strongly identify with their group require fewer economic incentives to cooperate (Van Vugt, 2009) and strong social identification can also buffer differences between individualist and collectivist members in terms of cooperation (Chen et al., 2007). Moreover, communication has an especially positive effect on cooperation when it is used to foster a common social identity (Adams et al., 2022). Finally, men, but not women, display a stronger identification with their group and consequently cooperate more if an intergroup comparison is salient (Van Vugt et al., 2007).

Next, incentives, social norms, trust, and anonymity each display five connections with other factors. Beginning with incentives, these are linked with social identification (as mentioned above), personality traits, trust, anonymity, and social status. Incentives can buffer the difference between pro-self and pro-social individuals by motivating pro-self individuals to increase their cooperation (Balliet et al., 2009). Moreover, when incentives are present, values and personality traits have less impact on cooperation (Schlösser et al., 2018; Hilbig et al., 2012). Incentives can also reduce individual trust in the motivation of other members to cooperate (Irwin et al., 2014) and in non-anonymous situations, cooperation is higher because there are more social incentives to cooperate (Rege and Telle, 2004). Finally, incentives work better in homogeneous groups: in mixed groups, punishment of defection is lower and this has a negative effect on cooperation (Drouvelis et al., 2021). Moving on to social norms, these are linked with social identification (as mentioned above), personality traits, anonymity, communication, and trust. Indeed, people with high collectivist values appear more sensitive to cooperative social norms than those with more individualist values (Chen et al., 2007). Moreover, in non-anonymous situations, people comply more with social norms (Rege and Telle, 2004), and the positive effect of communication on cooperation is partly explained by the fact that communication makes it possible to shape cooperative social norms (Bicchieri, 2002). Further, receiving feedback about the social norm of cooperation (e.g., regarding another participant's contribution) impacts the trust that participants have in each other (Irlenbusch et al., 2019). This brings us to the third factor with five connections: trust. It displays links with incentives and social norms (both explained above), social status, gender, and anonymity. Indeed, group heterogeneity is negatively correlated with in-group trust (Van Klingeren and De Graaf, 2021), and trust has more impact on women's cooperation than on men's (Irwin et al., 2015). Finally, in non-anonymous situations, individuals with lower levels of trust increase their cooperation (De Cremer et al., 2001). The fourth factor displaying five connections is anonymity. Besides the links with social norms, incentives, and trust explained directly above, anonymity is also linked with personality traits and communication. First, the difference in cooperation between anonymous and non-anonymous situations is greater among individuals who possess a high level of image concern (Christens et al., 2019). Second, communication can reduce anonymity in several ways, for example through promises to cooperate in a public way (Przepiorka and Diekmann, 2020) or through the exchange of reputational information by means of gossip (Feinberg et al., 2014).

Finally, two factors—specifically, social status and values/personality traits—display four links, whereas the two remaining factors—communication and gender—exhibit three links. All of these links have already been detailed above, except for that between social status and gender: here, social status can partly explain the gender differences observed in cooperative behavior (Sell and Kuipers, 2009).

## 4 Discussion

The present scoping review aimed to fulfill two research aims: namely, to identify factors impacting cooperation at (1) the individual level and (2) the organizational level. Based on our analysis of 135 articles, we identified a total of nine factors that enabled us to fulfill these aims. First, on the individual level, we found that strong social identification with the group and high trust in other group members both contribute to higher levels of cooperation. Values and personality traits also influence cooperation, and, in our review, we identified several specific effects: first, SVO has a very strong effect on cooperation, with pro-social individuals cooperating more than pro-self individuals. Political orientation also plays a role, with those on the left of the political spectrum generally contributing more than those on the right. Moreover, pro-environmental values (measured with the Schwartz Value Survey; Schwartz, 1992) and justice sensitivity are both positively correlated with cooperation. Concerning personality traits, individuals scoring high in self-control, Honesty-Humility (from the HEXACO model of personality; Ashton and Lee, 2007), and Agreeableness (from the Big Five personality model; Costa and McCrae, 1992) display more cooperation. Finally, two other individual factors—namely, gender and social status—also impact cooperation; however, the results for these factors did not enable clear conclusions about their effects. Second, on the organizational level, our results showed that the presence of incentives to cooperate, cooperative social norms, opportunities to communicate with others, and low anonymity all positively impact cooperation.

Moreover, our analysis showed that the nine factors we identified are interconnected. Analyzing these links enabled us to identify which factors are more connected to the others and, consequently, have more impact on cooperation. As shown in Figure 3, our results pointed to social identification as the most highly linked factor. The high relevance of social identification with respect to cooperation can be explained by the fact that people's identity also comprises their group affiliations. Consequently, the more they identify with a particular social group, the more important this group becomes to their identity and the more their personal interests align with those of the group (Tajfel, 1974). Social identification is also at the core of several theoretical models aimed at explaining collective behaviors, including the Social Identity Model of Collective Action (Van Zomeren et al., 2008) and the Social Identity Model of Pro-Environmental Action (Fritsche et al., 2018). Our analysis reinforces these models by highlighting social identification as a central determinant of individual cooperation. Nevertheless, social identification alone does not fully explain people's cooperative behavior. Indeed, both of the models mentioned above (SIMCA and SIMPEA) include additional determinants. Similarly, our results also highlighted other factors that follow close behind social identification in terms of number of links—namely, social norms, incentives, trust, and anonymity. In the context of commons, these findings suggest that, to cooperate, people not only need to identify with the group of commoners, but also need the following: guidance on how to behave (provided by social norms), motivation to act in the interest of the commons (provided by economic or social incentives), and trust in the fact that other commoners will also act for the collective good. Finally, knowing that their actions will not be anonymous is also central to increasing cooperation between commoners. Moreover, the five core factors we identified contribute to optimizing the effectiveness of the other factors. For instance, encouraging strong social identification with the group in combination with incentives can ensure that even individualist-minded people are motivated to cooperate. Moreover, fostering a “commons” social identity and increasing trust between commoners can buffer the negative impact of having a heterogeneous group in terms of social status. In conclusion, although every factor has a specific impact on cooperation, our analysis of the connections between the nine factors enabled us to identify those that are most central and highly linked, impacting cooperation both directly and indirectly.

Our scoping review also makes a significant contribution to theory development in the field of commons, with important implications for future research in real-life settings and for the design of behavioral interventions to improve cooperation. In the following, we will first discuss our results in the context of previous literature about commons. Second, we will detail how our results can shape future research in the field of the commons, and how they can be applied to motivate individuals to cooperate.

### 4.1 Implications for the development of commons theory

In general, our results fit with previous literature about commons and expand on it by providing new insights. First, our findings support Ostrom's (1990) principles for successful organization of commons^7^ and provide additional explanations as to why these principles work. We detail these links below and provide a summary in Table 4.

**Table 4:** Links between Ostrom's principles (1990) and our results.

**Ostrom's principles**	**Related individual factors**	**Related organizational factors**
Clearly defined boundaries	• Deciding who has and who has not access to the shared resource create an in- and out-group. Consequently, this strengthens commoners' social identification.	• To define who is and who is not part of the commons, commoners must know each-other, i.e., anonymity must be low. • Communication is essential to agree on the boundaries of the shared resource.
Congruence between rules and conditions of the resource	• Commoners' wealth can determine which share of the resource they can use, i.e., rules can be based on social status.	• Rules can be seen as social norms. • Communication between commoners enables to promptly adapt the rules to the resource's conditions.
Possibility for commoners to participate in the decision-making process	• In heterogeneous (i.e., with different social status) groups, inter-class conflicts can arise. Giving everybody the opportunity to contribute in creating the rules can mitigate these conflicts.	• Communication plays an important role in the decision-making process.
Monitoring and graduated sanctions systems	• Since both systems rely on incentives, they ensure that even individuals with a low social identification, or with a pro-self Social Value Orientation, will cooperate. • In order to give only a small fine for the first infraction, commoners must be confident that the person who broke the rules won't persist, i.e., they have to trust this person.	• With these systems commoners are accountable for their actions, i.e., there is less anonymity. • The systems rely on both social and material incentives.
Conflict-resolution mechanisms		• Conflicts arise when social norms are not respected. • Communication ensures that rules breakers can explain themselves, and therefore that cooperation do not drop.
Recognition of commoners' right to self-organize	• The legitimization of the group of commoners by the state can strengthen their social identification.	

Ostrom's first principle concerning the need for clearly defined boundaries relates to the aspect of anonymity that we identified in our review. In fact, commoners must know each other to define who can or cannot access the resource. Moreover, communication, as found in our review, is also important in order to reach an agreement on the exact boundaries of shared resources. Finally, this principle is also linked to the concept of social identity, since an in- and an out-group must exist for social identity to acquire salience and identification to occur. For this reason, even if Ostrom developed this principle on an organizational level, the secondary effect of creating a social identity for commoners can also help to improve cooperation. The second and third principles posit that rules must be adapted to the resource condition, and that commoners must be able to participate in modifying them. What Ostrom calls “rules” are very close to what we identified as social norms, and, once again, communication is essential to shape and adapt them according to the condition of the resource. Moreover, rules can be linked to social status: for instance, in Töbler's meadows described in the introduction, commoners' capacity to feed the cows during winter determines the number of cows they are allowed to send to the meadow in summer. This means that wealthier people can use a greater share of the common resource. As our results highlighted, differences in access to resources can create inter-class conflicts between high- and low-status classes, especially due to intra-class solidarity among low-status members. However, the fact that every commoner can contribute to create and modify the rules can mitigate this negative effect of social status, insofar as everyone perceives the rules are fair. Ostrom's fourth principle concerns systems of monitoring, underscoring the importance of non-anonymity for cooperation. In fact, monitoring discourages free-riding because commoners know that their actions can be observed by others. Moreover, such systems also rely on incentives, both social and material: first, a monitor who discovers an infraction gains prestige by being a good member of the commons, while the rule-breaker loses it. Second, the monitor can often keep a portion of the violator's harvest, thus providing the former with personal material gain. As our results showed, incentives have a bigger effect on people who are not intrinsically motivated to cooperate. Thus, this system ensures that people with a weaker social identification, or with values or personality traits which don't encourage cooperation, are motivated to cooperate. The fifth principle (a graduated sanction system) is closely linked with the preceding one, and relating with incentives. Moreover, graduated sanctions (i.e., small for the first infraction and gradually higher for subsequent infractions) only function when commoners trust each other. In such cases, commoners will agree to small fines for first-time infractions because they trust that violators will not continue to break the rules. Furthermore, Ostrom's sixth principle concerning mechanisms for conflict resolution relies on communication, which enables commoners to clarify their positions and find solutions. This principle is also linked to social norms, since conflicts arise when social norms are violated or misinterpreted: the fact that violators have the possibility to explain themselves prevent the rules from being perceived as unfair, which would harm cooperation. Finally, the seventh principle posits that the state must recognize the right of commoners to self-organize. This recognition from the state legitimizes the group of commoners and, consequently, can increase their social identification.

In conclusion, Ostrom's (1990) design principles closely relate to the factors identified in our scoping review. This is not surprising with respect to organizational factors given that, in her work, Ostrom viewed commons as organizations around a shared resource, with little focus on commoners' individual characteristics. Interestingly, however, the individual factors we identified also partly fit in Ostrom's framework, albeit less directly than the organizational factors. The importance of considering individual factors when studying cooperation in commons is also demonstrated by more recent commons literature that has started to adopt an individual approach. This new perspective has identified several individual factors that could impact cooperation, such as trust and reciprocity (Janssen, 2015). Poteete et al. (2010) also identified heterogeneity of access to the resource as a structural variable that usually hinders cooperation. These results are also confirmed by Agrawal et al. (2023), who, in their review, highlighted that a heterogeneous group of commoners is less likely to successfully manage the shared resource. Third, Agrawal et al. (2023) also identified commoners' gender as a factor influencing cooperation, focusing on gender inequalities with respect to the access to the resource and governance tasks. Finally, recent studies have also demonstrated that the personal level of psychological ownership, i.e., the feeling of ownership of the shared resource, is a crucial determinant of individual cooperation (Peck et al., 2021; Ambuehl et al., 2022). In this way, our emphasis on individual factors impacting cooperation in commons resonates with this new current of commons literature. Interestingly, however, the central factor we identified in the current review—social identification—is, to the best of our knowledge, rarely investigated in commons literature.

To summarize, our work has two main implications with respect to the existing literature about commons. First, it shows that a classical work of commons literature—namely Ostrom's seminal book (Ostrom, 1990)—can be confirmed by findings coming from other disciplines. Second, it shows that social identification, a factor seldom considered in commons literature, plays a central role in motivating individuals to cooperate. This result shows that social psychology and behavioral economics can make a significant contribution to study of the commons. In the following section, we will discuss the practical implications of our findings for future research, as well as for policymaking and behavioral interventions.

### 4.2 Implications for future research and behavioral interventions

Our scoping review has important implications for future field research in and on commons, as well as for the design of behavioral interventions aimed at improving cooperation in commons. First, it is important to note that, with the current scoping review, we did not fully achieve our goal to integrate literature about commons and about social dilemma experiments. Indeed, despite our efforts to represent these literature strands equally via the search keywords, the articles included in our review were mostly experimental studies with a laboratory setting. This further confirms that individual cooperation has not been extensively investigated in commons literature, and that adopting an individual-centered approach can provide new insights into cooperation in commons. However, a consequence of this underrepresentation of commons literature in the current review is that most of the results we identified have not been demonstrated in real-world settings. Future research could therefore address this gap—for example by developing experimental studies in real-life commons—in order to (1) gain a better understanding of the causes of cooperation among commoners and (2) assess the validity of laboratory studies' findings in real-life commons. Our scoping review offers a foundation for the implementation of such studies, by highlighting a set of determinants that could impact cooperation in commons. Real-world studies on individual cooperation in commons could significantly contribute to the development of this field by testing in practice the results obtained in laboratory settings. Indeed, although commons and social dilemmas share the same basic conflict between personal and collective interests, lab-based social dilemma experiments cannot precisely reproduce the social dynamics of actual commons (Cole and Grossman, 2010; Henry and Dietz, 2011). Our review also provides a hierarchical classification of identified factors, ranked according to their centrality. This analysis highlighted the importance of social identity for cooperation in commons; future research could test these results with a more quantitative approach. This would make it possible to expand the literature about commons, which seldom considers social identification. Finally, in the current scoping review, we could not draw a clear conclusion about how gender and social status impact cooperation, thus leaving an open question about their effects. Consequently, future studies could address these issues and try to provide more definitive answers to these questions.

Second, our review provides interesting insights for the development of interventions aimed at improving cooperation around common resources. The five central factors we identified—social identification, social norms, incentives, trust, and anonymity—are important levers to encourage people to cooperate. Thus, according to our findings, when designing interventions to improve individuals' cooperation it seems important to take the following aspects into account: (1) to recognize the social group of reference—i.e., *who* is sharing the resource—and foster a common social identity so that commoners can develop a feeling of belonging and perceive their personal interest as aligned with that of the group; (2) to communicate a cooperative social norm, for example by demonstrating that other commoners are cooperating; (3) to provide incentives, for example in the form of social approval, as further motivation to cooperate; (4) to increase commoners' trust in each other; (5) to ensure that commoners' actions are accountable. Moreover, the other factors we identified can also be used to encourage individuals to cooperate. Providing commoners with the possibility to communicate—such as in a social event—would greatly benefit cooperation, for example. Our results also highlighted the importance of considering the group composition when developing behavioral interventions, as it can greatly impact cooperation. For instance, improving commoners' trust in each other is especially important in groups that are relatively heterogeneous in terms of people's social status. Similarly, in cases where most commoners do not have values or personality traits favoring cooperation, it is likely better to emphasize economic incentives to cooperate.

Finally, individual cooperation is also impacted by several contextual factors that should be considered when applying the results of the current scoping review—whether in planning research in real-world commons or in designing interventions to improve cooperation in commons. First, cultural context plays a major role in cooperative situations. Indeed, in South American, African, and Asian cultures, people generally display higher levels of collectivism, whereas people in European and North American cultures are more geared toward individualism (Triandis, 1989; Darwish and Huber, 2003). This has an impact on the extent to which people define themselves as a part of a group: in collectivist cultures, people more readily perceive themselves as belonging to a social group in comparison with individualist cultures (Trafimow et al., 1991). There are also cultural differences in individual levels of generalized trust, and these differences impact people's displays of cooperation and solidarity or lack thereof (Kuwabara et al., 2014). On a more practical level, the efficiency of political institutions can also vary according to cultural norms and values: for example, several studies have shown that individuals comply more readily with laws and rules that have been arrived at democratically. However, in strongly authoritarian cultures such as China, individuals also display high levels of obedience to authorities and comply more readily with laws imposed in a top-down manner (Vollan et al., 2013). Moreover, culture can affect the impact of the factors identified in the present review: for instance, punishment is more effective in enhancing cooperation in cultures with a stronger norm of civic cooperation, since there is less anti-social punishment in these cultures (Herrmann et al., 2008). In conclusion, cultural factors greatly impact the way in which individuals see themselves, interact, cooperate, and comply with social norms.

Secondly, the way in which the cooperative interaction is presented, or framed, can also influence cooperation. This is known as the “framing effect”, and it has mainly been studied in laboratory experiments: if the instructions of a cooperation-oriented game highlight the positive externalities of cooperative behavior, cooperation tends to be higher than if the negative externalities of selfish behavior are highlighted. This suggests that, generally, the feeling of doing something good provides greater motivation to cooperate than concerns about (not) doing something bad (Andreoni, 1995). However, more recent studies suggest that the framing effect is stronger for individualist-minded people than collectivist-minded people (Park, 2000), and that the trend does not hold in every culture (Goerg and Walkowitz, 2010).

Third, the size of the stakes also impacts cooperation: an experiment showed that participants cooperated more in a hypothetical game (i.e., when they know that they will not be paid according to their income at the end of the game) than in an incentivized one (Lönnqvist et al., 2011). Similarly, a meta-analysis found that the size of the stakes is negatively correlated with the generosity of participants (Larney et al., 2019). Finally, in a review of existing experiments, Karagözoglu and Urhan (2017) reported some cases in which higher stakes were related to more selfish behavior, though they concluded that there was not enough evidence to draw a clear conclusion on this effect. Taken together, this evidence suggests that individuals cooperate less when the consequences of the cooperative interaction are weightier.

In conclusion, when applying the results of the current review to real-life commons, it is important to consider the three contextual elements described above because they could (1) impact how individuals perceive the situation and behave and (2) moderate the effects of the factors identified in our review on cooperation.

### 4.3 Limitations and future perspectives

Our scoping review has several potential limitations. First, the addition of other key search terms—such as “participation” or “common pool resource”—in combination with those we used could have led to the inclusion of other relevant papers (e.g., Ambuehl et al., 2022). Future reviews might include these additional terms in order to expand on our results. Second, while the quality of the works included in this scoping review is generally satisfactory, a few of the papers come from journals with somewhat lower standards of quality (see Appendix D for our complete quality assessment of included papers). Future work should apply a quality metric for the journals/papers as an inclusion or exclusion criterion, in order to increase the reliability of the review's results. Finally, our scoping review is subject to a general cultural/geographic bias in the existing scientific literature, namely that studies conducted in Western societies are overrepresented. Indeed, two-thirds of the papers we identified involved studies conducted in Europe or North America, and the sixteen reviews we included were also mostly based on studies from Western countries. Consequently, our findings and their implications primarily apply to Western societies and cannot be generalized to others. Further research should investigate the dynamics of individual cooperation in non-Western cultures to reach a more global understanding on this topic.

In conclusion, our scoping review has several useful implications for the development of commons theory, as well as for future research on commons and for interventions aimed at improving cooperation. However, it is not without limitations, and thus further research is needed to address the weaknesses of the current review and expand our knowledge of how cooperation in commons can and does function.

## 5 Conclusions

Our scoping review sought to identify which individual and organizational factors could influence cooperation in commons. Our results showed that the following factors influence cooperation on the individual level: gender, social status, social identification, values and personality traits, and trust. On the organizational level, by contrast, we identified the following factors that influence cooperation: incentives, communication, social norms, and anonymity. In this way, our scoping review expands on classical commons literature, demonstrating that the field can be enhanced by considering individual characteristics. Moreover, it introduces social identification as a central determinant of individual cooperation, although further studies are needed to confirm and expand these results in real-world commons settings.

In addition to improving understanding of these specific factors, the present review also strengthened the argument that people are indeed capable of cooperating in commons settings. This contradicts Hardin's (1968) assumption that rational individuals are too self-interested to cooperate in a situation where individual and group interests are at odds. Our results are consistent with more recent literature, including theories such as the Greed Efficiency Fairness Hypothesis (Wilke, 1991), and the Humanistic Rational Choice Theory (DeCaro et al., 2021), all of which posit that cooperation is possible. Finally, this review extends efforts to link social dilemma and commons literature. In fact, previous studies using the same approach focused either on a specific commons (e.g., Hartl and Hofmann, 2022) or on a specific discipline (Kopelman et al., 2002). We have extended these results by studying cooperation in general, without excluding any scientific field.

In conclusion, the current review makes important contributions to the study of cooperation within commons by proposing several explanatory factors of individual cooperation. This topic is of great importance today, since commons represent a possible way to mitigate the detrimental environmental and social effects of mainstream social organization. Therefore, they could be a way to attain what Raworth defined a “safe and just space for humanity” (Raworth, 2012, p. 4).

## Footnotes

^1^The term “commons” is both singular and plural (Bollier, 2014). For the difference between the singular forms “common” and “commons”, see Feinberg et al. (2021).

^2^Since the eighth principle is not relevant for the current paper, it will not be detailed.

^3^While only the first author was responsible for this procedure, every stage of the selection process, including final selection, was discussed among the whole consortium of authors. The reason for having one person responsible for the selection process was to avoid any potential inter-rater bias in the selection of papers.

^4^Studies where at least one variable is manipulated by researchers belong to the category “experimental studies”, whereas the category “correlative studies” includes papers using quantitative analysis, but without manipulations.

^5^The use of authentic money facilitated real consequences for decisions taken in the laboratory. For this reason, even studies that initially used fictive money often informed participants that their final sum would be converted into real money at the end of the experiment.

^6^In this section “incentives” is used when results apply to both punishments and rewards. Otherwise, the kind of system is explicitly mentioned.

^7^As mentioned in our introduction, the eighth principle concerns more complex cases and, for this reason, it will not be discussed here.
