# Supplementary material for: Exploring individual and organizational factors influencing cooperation in commons: a scoping review
Source: Front Psychol. 2025 Jun 3;16:1465057. doi: 10.3389/fpsyg.2025.1465057 (PMC12170531; doi:10.3389/fpsyg.2025.1465057)
Supplement: Supplementary file 1 [file Supplementary_file_1.docx]

**Appendix A**

**Research protocol established by the authors before the search phase**

- Research methodology
  - Keywords

Commons, social dilemmas, cooperation, conflict, reciprocity, trust

Combinations:

- commons AND social dilemma
- commons AND cooperation
- commons AND conflicts
- commons AND reciprocity
- commons AND trust
- social dilemma AND cooperation
- social dilemma AND conflicts
- social dilemma AND reciprocity
- social dilemma AND trust
- public good AND cooperation
- public good AND conflicts
- public good AND reciprocity
- public good AND trust
- common resource AND cooperation
- common resource AND conflicts
- common resource AND reciprocity
- common resource AND trust
  - Search engines
- Scopus
- Web of Science
- ScienceDirect
  - Procedure
    - 1. Insert every combination of keywords in every search engine.
      2. Download the metadata (title, authors, year, DOI) of all results.
      3. Merge all the .csv files obtained.
      4. Delete the duplicates
- Inclusion criteria
  - Main criteria
- Cooperation as main outcome variable
- Test of the impact of one or more factor on cooperation
  - Methodological criteria
- English
- Adult human participants
- Screening process
  - - 1. Read the titles and exclude the papers that are clearly off-topic.
      2. Read the abstracts of the remaining papers and select the ones that seem to fit to the criteria. If in doubt, select the paper.
      3. Read the full texts and select the ones that fit to the criteria.
