# Supplementary material for: Exploring individual and organizational factors influencing cooperation in commons: a scoping review
Source: Front Psychol. 2025 Jun 3;16:1465057. doi: 10.3389/fpsyg.2025.1465057 (PMC12170531; doi:10.3389/fpsyg.2025.1465057)
Supplement: Supplementary file 2 [file Supplementary_file_2.docx]

**Appendix B**

**Overview of the 105 selected papers, and of how they meet the inclusion criteria**

The following table provides an overview of the 105 papers included in the current scoping review. Each paper is categorized in terms of setting and context, and methodology (see also Table 1 for a summary). Moreover, for each paper we specified how it met our two main inclusion criteria (IC), namely (1) to measure cooperation as main outcome variable and (2) to test the impact of at least one factor on cooperation.

| Author(s) and date | Title | Setting and context | Methodology | IC1: cooperation as main outcome variable | IC2: main factor impacting cooperation |
| --- | --- | --- | --- | --- | --- |
| Adams et al. (2022) | Verbal interaction in a social dilemma | Lab-based social dilemma | Qualitative | Fictive money invested in a public good | Communication |
| Aksoy (2019) | Crosscutting circles in a social dilemma: Effects of social identity and inequality on cooperation | Lab-based social dilemma | Experimental | Dichotomous choice between cooperation and defection (each associated with different payoff for the self and for the other participants) | Social status |
| Almeida (2023) | Punishment credibility and cooperation in public good games | Lab-based social dilemma | Experimental | Fictive money invested in a public good | Incentives |
| Ambrus and Greiner (2012) | Imperfect public monitoring with costly punishment: An experimental study | Lab-based social dilemma | Experimental | Fictive money invested in a public good | Incentives |
| Arora et al. (2016) | Acting for the Greater Good: Identification with Group Determines Choices in Sequential Contribution Dilemmas: Group Identity and Sequential Social Dilemmas | Lab-based social dilemma | Experimental | Money invested in a public good | Social identification |
| Arora et al. (2012) | To cooperate or not to cooperate: Using new methodologies and frameworks to understand how affiliation influences cooperation in the present and future | Lab-based social dilemma | Experimental | Dichotomous choice between cooperation and defection (each associated with different payoff for the self and for the other participants) | Social norms |
| Balliet (2010) | Communication and Cooperation in Social Dilemmas: A Meta-Analytic Review | Lab-based social dilemma | Meta-analysis | Only studies measuring cooperation in a social dilemma setting were included | Communication |
| Balliet and Van Lange (2013a) | Trust, conflict, and cooperation: A meta-analysis | Lab-based social dilemma | Meta-analysis | Only studies measuring cooperation in a social dilemma setting were included | Trust |
| Balliet and Van Lange (2013b) | Trust, punishment, and cooperation across 18 societies: A meta-analysis | Lab based social dilemma | Meta-analysis | Only studies measuring cooperation in a social dilemma setting were included | Incentives |
| Balliet et al. (2011) | Sex differences in cooperation: A meta-analytic review of social dilemmas | Lab-based social dilemma | Meta-analysis | Only studies measuring cooperation in a social dilemma setting were included | Gender |
| Balliet et al. (2011) | Reward, punishment, and cooperation: A meta-analysis | Lab-based social dilemma | Meta-analysis | Only studies measuring cooperation in a social dilemma setting were included | Incentives |
| Balliet et al. (2009) | Social Value Orientation and cooperation in social dilemmas: A meta-analysis | Lab-based social dilemma | Meta-analysis | Only studies measuring cooperation in a social dilemma setting were included | Values and personality traits |
| Banerjee (2024) | The effect of heterogeneity and risk on co-operation: experimental evidence | Lab-based social dilemma | Experimental | Fictive money invested in a public good | Social status |
| Bardsley and Sausgruber (2005) | Conformity and reciprocity in public good provision | Lab-based social dilemma | Experimental | Fictive money invested in a public good | Social norms |
| Barrero-Amórtegui and Maldonado (2021) | Gender composition of management groups in a conservation agreement framework: Experimental evidence for mangrove use in the Colombian Pacific | Real-life commons | Experimental | Extraction from the shared resource, investment for maintaining the shared resource | Gender |
| Baum et al. (2012) | Cooperation due to cultural norms, not individual reputation | Lab-based social dilemma | Experimental | Fictive money invested in a public good | Communication |
| Bechtel and Scheve (2017) | Who cooperates? Reciprocity and the causal effect of expected cooperation in representative samples | Lab-based social dilemma | Correlational | Fictive money invested in a public good | Trust |
| Bicchieri (2002) | Covenants without Swords: Group Identity, Norms, and Communication in Social Dilemmas | Lab-based social dilemma | Literature review | Actions for the collective interest | Communication |
| Bilancini et al. (2022) | Social value orientation and conditional cooperation in the online one-shot public goods game | Lab-based social dilemma | Experimental | Fictive money invested in a public good | Values and personality traits |
| Böhm and Rockenbach (2013) | The inter-group comparison – intra-group cooperation hypothesis: Comparisons between groups increase efficiency in public goods provision | Lab-based social dilemma | Experimental | Fictive money invested in a public good | Social identification |
| Butz and Harbring (2021) | The Effect of Disclosing Identities in a Socially Incentivized Public Good Game | Lab-based social dilemma | Experimental | Fictive money invested in a public good | Anonymity |
| Camera et al. (2020) | Do economic inequalities affect long-run cooperation and prosperity? | Lab-based social dilemma | Experimental | Dichotomous choice between cooperation and defection (each associated with different payoff for the self and for the other participants) | Social status |
| Chaudhuri and Paichayontvijit (2017) | On the long-run efficacy of punishments and recommendations in a laboratory public goods game | Lab-based social dilemma | Experimental | Fictive money invested in a public good | Incentives |
| Chaudhuri et al. (2002) | Cooperation in social dilemmas, trust and reciprocity | Lab-based social dilemma | Experimental | Dichotomous choice between cooperation and defection (each associated with different payoff for the self and for the other participants) | Trust |
| Chen (2022) | Carrots and sticks: new evidence in public goods games with heterogeneous groups | Lab-based social dilemma | Experimental | Fictive money invested in a public good | Incentives |
| Chen et al. (2007) | When does group norm or group identity predict cooperation in a public goods dilemma? The moderating effects of idiocentrism and allocentrism | Lab-based commons dilemma | Experimental | Number of extra hours participant were willing to (fictitiously) invest during the weekend | Values and personality traits |
| Chen et al. (2009) | Unintended consequences of cooperation inducing and maintaining mechanisms in public goods dilemmas: Sanctions and moral appeals | Lab-based commons dilemma | Experimental | Fictive money invested in a public good | Incentives |
| Christens et al. (2019) | Identification of individuals and groups in a public goods experiment | Lab-based commons dilemma | Experimental | Fictive money invested in a public good | Anonymity |
| Cinyabuguma et al. (2005) | Cooperation under the threat of expulsion in a public goods experiment | Lab-based commons dilemma | Experimental | Fictive money invested in a public good | Incentives |
| Colman et al. (2018) | Persistent cooperation and gender differences in repeated Prisoner’s Dilemma games: Some things never change | Lab-based social dilemma | Experimental | Dichotomous choice between cooperation and defection (each associated with different payoff for the self and for the other participants) | Gender |
| De Cremer and Leonardelli (2003) | Cooperation in social dilemmas and the need to belong: The moderating effect of group size | Lab-based commons dilemma | Experimental | Fictive money invested in a public good | Social identification |
| De Cremer et al. (2001) | ‘The less I trust, the less I contribute (or not)?’ The effects of trust, accountability and self‐monitoring in social dilemmas | Lab-based commons dilemma | Experimental | Fictive money invested in a public good | Trust |
| De Kwaadsteniet et al. (2008) | ‘How Many of Us Are There?’: Group Size Uncertainty and Social Value Orientations in Common Resource Dilemmas | Lab-based social dilemma | Experimental | Resource taken from a common pool | Values and personality traits |
| Dorrough and Glöckner (2019) | A cross-national analysis of sex differences in prisoner’s dilemma games | Lab-based social dilemma | Experimental | Fictive money given to the interaction partner | Gender |
| Dorrough et al. (2015) | The development of ingroup favoritism in repeated social dilemmas | Lab-based social dilemma | Experimental | Fictive money given to the interaction partner | Social identification |
| Drouvelis et al. (2021) | Cooperation in a fragmented society: Experimental evidence on Syrian refugees and natives in Lebanon | Lab-based commons dilemma | Experimental | Fictive money invested in a public good | Social status |
| Egas and Riedl (2005) | The economics of altruistic punishment and the maintenance of cooperation | Lab-based social dilemma | Experimental | Fictive money invested in a public good | Incentives |
| Emonds et al. (2011) | Comparing the neural basis of decision making in social dilemmas of people with different social value orientations, a fMRI study | Lab-based social dilemma | Experimental | Dichotomous choice between cooperation and defection (each associated with different payoff for the self and for the other participants) | Values and personality traits |
| Fehr and Gätcher (2000) | Cooperation and punishment in public goods experiments | Lab-based commons dilemma | Experimental | Fictive money invested in a public good | Incentives |
| Feinberg et al. (2014) | Gossip and Ostracism Promote Cooperation in Groups | Lab-based social dilemma | Experimental | Fictive money invested in a public good | Communication |
| Feinberg et al. (2012) | The virtues of gossip: Reputational information sharing as prosocial behavior | Lab-based social dilemma | Experimental | Fictive money invested in a public good | Communication |
| Fosgaard et al. (2019) | Cooperation, framing, and political attitudes | Lab-based social dilemma | Experimental | - Fictive money invested in a public good - Fictive money taken from a common pool | Values and personality traits |
| Franzen et al. (2019) | Governing the Commons: Why Self-Administered Farm Outlets Flourish in Switzerland | Real-life social dilemma | Correlational | Having a self-administered outlet | Trust |
| Gätcher et al. (2004) | Trust, voluntary cooperation, and socio-economic background: survey and experimental evidence | Lab-based social dilemma | Correlational | Fictive money invested in a public good | Trust |
| Ghate et al. (2013) | Cultural norms, cooperation, and communication: Taking experiments to the field in indigenous communities | Real-life commons | Experimental | Resource taken from a common pool | Communication |
| Giardini et al. (2021) | Gossip and competitive altruism support cooperation in a Public Good game | Lab-based social dilemma | Experimental | Fictive money invested in a public good | Communication |
| Gomez-Ruiz and Sánchez-Expósito (2020) | The impact of team identity and gender on free-riding responses to fear and cooperation sustainability | Lab-based social dilemma | Experimental | Dichotomous choice between cooperation and defection (each associated with different payoff for the self and for the other participants) | Gender |
| Grechenig et al. (2010) | Punishment despite reasonable doubt—A public goods experiment with sanctions under uncertainty | Lab-based social dilemma | Experimental | Fictive money invested in a public game | Incentives |
| Grünhage and Reuter (2022) | Political orientation is associated with behavior in public-goods- and trust-games | Lab-based social dilemma | Correlational | Fictive money invested in a public good | Values and personality traits |
| Hassan et al. (2023) | Incentivizing cooperation against a norm of defection: experimental evidence from Egypt | Lab-based social dilemma | Experimental | Fictive money invested in a public good | Social norms |
| Haucap et al. (2024) | Gender and cooperation in the presence of negative externalities | Lab-based social dilemma | Experimental | Fictive money invested in a public good | Gender |
| Herrmann et al. (2008) | Antisocial punishment across societies | Lab-based social dilemma | Experimental | Fictive money invested in a public good | Incentives |
| Hilbig et al. (2012) | Personality, punishment and public goods: Strategic shifts towards cooperation as a matter of dispositional Honesty–Humility | Lab-based social dilemma | Experimental | Fictive money invested in a public good | Values and personality traits |
| Hilbig et al. (2018) | Lead us (not) into temptation: Testing the motivational mechanisms linking Honesty–Humility to cooperation | Lab-based social dilemma | Experimental | Dichotomous choice between cooperation and defection (each associated with different payoff for the self and for the other participants) | Values and personality traits |
| Hill and Gurven (2004) | Economic experiments to examine fairness and cooperation among the Ache Indians of Paraguay | Lab-based social dilemma | Experimental | Fictive money invested in a public good | Anonymity |
| Hoenow and Pourviseh (2024) | Intragroup communication in social dilemmas: An artefactual public good field experiment in small-scale communities | Lab-based social dilemma | Experimental | Fictive money invested in a public good | Communication |
| Hopthrow and Hulbert (2005) | The Effect of Group Decision Making on Cooperation in Social Dilemmas | Lab-based social dilemma | Experimental | Dichotomous choice between cooperation and defection (each associated with different payoff for the self and for the other participants) | Communication |
| Irlenbusch et al. (2019) | Designing feedback in voluntary contribution games: the role of transparency | Lab-based social dilemma | Experimental | Fictive money invested in a public good | Trust |
| Irwin et al. (2015) | Gender, trust and cooperation in environmental social dilemmas | Real-life social dilemma | Correlational | Pro-environmental measures adopted in the private sphere and sustained through political decisions | Gender |
| Irwin et al. (2014) | The Detrimental Effects of Sanctions on Intragroup Trust: Comparing Punishments and Rewards | Lab-based social dilemma | Experimental | Fictive money invested in a public good | Incentives |
| Jackson (2011) | Intragroup cooperation as a function of group performance and group identity | Lab-based social dilemma | Experimental | Fictive money invested in a public good | Social identification |
| Jackson (2012) | Reactions to a social dilemma as a function of intragroup interactions and group performance | Lab-based social dilemma | Experimental | Fictive money invested in a public good | Social identification |
| Jacquet et al. (2011) | Shame and honour drive cooperation | Lab-based social dilemma | Experimental | Fictive money invested in a public good | Incentives |
| Janssen et al. (2014) | The effect of constrained communication and limited information in governing a common resource | Lab-based commons dilemma | Experimental | Resource taken from a common pool | Communication |
| Jin et al. (2024) | Institutions and cooperation: A meta-analysis of structural features in social dilemmas | Lab-based social dilemma | Meta-analysis | - Fictive money invested in a public good - Dichotomous choice between cooperation and defection (each associated with different payoff for the self and for the other participants)  - Resource taken from a common pool" | Review of several factors impacting cooperation |
| Jiang et al. (2021) | Reducing the bystander effect via decreasing group size to solve the collective-risk social dilemma | Lab-based social dilemma | Experimental | Fictive money invested in a public good | Anonymity |
| Kieslich and Hilbig (2014) | Cognitive conflict in social dilemmas: An analysis of response dynamics | Lab-based social dilemma | Experimental | Dichotomous choice between cooperation and defection (each associated with different payoff for the self and for the other participants) | Values and personality traits |
| Kingsley (2016) | Endowment heterogeneity and peer punishment in a public good experiment: Cooperation and normative conflict | Lab-based social dilemma | Experimental | Fictive money invested in a public good | Incentives |
| Kocher et al. (2015) | The role of beliefs, trust, and risk in contributions to a public good | Lab-based social dilemma | Correlational | Fictive money invested in a public good | Trust |
| Kocher et al. (2017) | Strong, bold, and kind: self-control and cooperation in social dilemmas | Lab-based social dilemma | Correlational | Fictive money invested in a public good | Values and personality traits |
| Koessler, Ortiz-Riomalo, et al. (2021) | Structuring Communication Effectively—The Causal Effects of Communication Elements on Cooperation in Social Dilemmas | Lab-based social dilemma | Experimental | Fictive money invested in a public good | Communication |
| Koessler, Page, et al. (2021) | Public cooperation statements | Lab-based social dilemma | Experimental | Fictive money invested in a public good | Anonymity |
| Kramer and Brewer (1984) | Effects of group identity on resource use in a simulated commons dilemma | Lab-based commons dilemma | Experimental | Resource taken from a common pool | Social identification |
| Kumakawa (2013) | Evaluating others’ behavior: a public-good experiment with ex-post communication | Lab-based social dilemma | Experimental | Fictive money invested in a public good | Communication |
| Lavallee et al. (2024) | A conservation orientation in commons dilemmas | Lab-based commons dilemma | Experimental | Resource taken from a common pool | Social norms |
| Liu and Li (2009b) | Contextualized self: When the self runs into social dilemmas | Lab-based social dilemma | Experimental | Fictive money invested in a public good | Values and personality traits |
| Lönnqvist et al. (2025) | Ideological constraint and behavioral consistency—A person-centered approach to political attitudes and Public Goods Games behavior | Lab-based social dilemma | Correlational | Fictive money invested in a public good | Values and personality traits |
| Lu et al. (2019) | True versus strategic fairness in a common resource dilemma: Evidence from the dual‐process perspective | Lab-based social dilemma | Experimental | Resource taken from a common pool | Values and personality traits |
| Lübke (2021) | The Climate Change Dilemma: How Cooperation Beliefs Influence Energy Conservation Behavior | Real-life social dilemma | Correlational | Energy conservation behaviors | Trust |
| Ma et al. (2024) | High level of self-disclosure on SNSs facilitates cooperation: A serial mediation model of psychological distance and trust | Lab-based social dilemma | Experimental | Dichotomous choice between cooperation and defection (each associated with different payoff for the self and for the other participants) | Anonymity |
| Maier-Rigaud et al. (2010) | Ostracism and the provision of a public good: experimental evidence | Lab-based social dilemma | Correlational | Fictive money invested in a public good | Incentives |
| Malthouse et al. (2023) | When fairness is not enough: The disproportionate contributions of the poor in a collective action problem | Lab-based social dilemma | Experimental | Fictive money invested in a public good | Social status |
| Milinski and Rockenbach (2012) | On the interaction of the stick and the carrot in social dilemmas | Lab-based social dilemma | Literature review | Actions for the collective interest | Incentives |
| Mosler (1993) | Self-dissemination of environmentally-responsible behavior: The influence of trust in a commons dilemma game | Lab-based commons dilemma | Experimental | Resource taken from a common pool | Anonymity |
| Mulder et al. (2006) | Undermining trust and cooperation: The paradox of sanctioning systems in social dilemmas | Lab-based social dilemma | Experimental | Fictive money invested in a public good | Incentives |
| Nelissen and Mulder (2013) | What makes a sanction “stick”? The effects of financial and social sanctions on norm compliance | Lab-based social dilemma | Experimental | Fictive money invested in a public good | Incentives |
| Nockur and Pfattheicher (2020) | Fostering sustainable behavior through group competition | Lab-based commons dilemma | Experimental | Resource taken from a common pool | Social identification |
| Nockur et al. (2021) | Different punishment systems in a public goods game with asymmetric endowments | Lab-based commons dilemma | Experimental | Fictive money invested in a public good | Incentives |
| Noonan et al. (2016) | Characteristics of Voluntary Behavior in the Neighborhood Commons: The Case of Dog Parks | Real-life commons | Correlational | Voluntary and philanthropic activities | Social identification |
| Noussair et al. (2024) | The role of emotions in public goods games with and without punishment opportunities | Lab-based social dilemma | Experimental | Fictive money invested in a public good | Incentives |
| Oyediran et al. (2018) | Cooperation and optimism in a social dilemma | Lab-based social dilemma | Experimental | Fictive money invested in a public good | Trust |
| Peng and Fan (2023) | Incomplete punishment networks, heterogeneity, and cooperation in public good experiments | Lab-based social dilemma | Experimental | Fictive money invested in a public good | Incentives |
| Peshkovskaya et al. (2019) | Gender effects and cooperation in collective action: A laboratory experiment | Lab-based social dilemma | Experimental | Dichotomous choice between cooperation and defection (each associated with different payoff for the self and for the other participants) | Gender |
| Peshkovskaya et al. (2017) | Do Women Socialize Better? Evidence from a Study on Sociality Effects on Gender Differences in Cooperative Behavior’ | Lab-based social dilemma | Experimental | Dichotomous choice between cooperation and defection (each associated with different payoff for the self and for the other participants) | Gender |
| Pfattheicher et al. (2018) | The Advantage of Democratic Peer Punishment in Sustaining Cooperation within Groups | Lab-based social dilemma | Experimental | Fictive money invested in a public good | Incentives |
| Probst et al. (1999) | Cultural Values in Intergroup and Single-Group Social Dilemmas | Lab-based social dilemma | Experimental | Dichotomous choice between cooperation and defection (each associated with different payoff for the self and for the other participants) | Values and personality traits |
| Przepiorka and Diekmann (2020) | Binding Contracts, Non-Binding Promises and Social Feedback in the Intertemporal Common-Pool Resource Game | Lab-based social dilemma | Experimental | Fictive money invested in a public good | Anonymity |
| Puurtinen and Mappes (2009) | Between-group competition and human cooperation | Lab-based social dilemma | Experimental | Fictive money invested in a public good | Social identification |
| Ramalingam and Stoddard (2024a) | Does reducing inequality increase cooperation? | Lab-based social dilemma | Experimental | Fictive money invested in a public good | Social status |
| Ramalingam and Stoddard (2024b) | Inequality reduction and cooperation: Injection of additional resources | Lab-based social dilemma | Experimental | Fictive money invested in a public good | Social status |
| Rand et al. (2009) | Positive interactions promote public cooperation | Lab-based social dilemma | Experimental | Fictive money invested in a public good | Incentives |
| Rege and Telle (2004) | The impact of social approval and framing on cooperation in public good situations | Lab-based social dilemma | Experimental | Fictive money invested in a public good | Social norms |
| Rockenbach and Wolff (2019) | The Dose Does it: Punishment and Cooperation in Dynamic Public-Good Games | Lab-based social dilemma | Experimental | Fictive money invested in a public good | Incentives |
| Romano et al. (2016) | On the role of group size in social dilemmas | Lab-based social dilemma | Literature review | Actions for the collective interest | Group size |
| Rompf et al. (2017) | Institutional trust and the provision of public goods: When do individual costs matter? The case of recycling | Real-life social dilemma | Correlational | Recycling behaviors | Trust |
| Schlösser et al. (2018) | Justice Sensitivity and Cooperation Dynamics in Repeated Public Good Games | Lab-based social dilemma | Experimental | Fictive money invested in a public good | Values and personality traits |
| Sell and Kuipers (2009) | A Structural Social Psychological View of Gender Differences in Cooperation | Lab-based social dilemma | Literature review | Actions for the collective interest | Gender |
| Simpson (2003) | Sex, Fear, and Greed: A Social Dilemma Analysis of Gender and Cooperation | Lab-based social dilemma | Experimental | Dichotomous choice between cooperation and defection (each associated with different payoff for the self and for the other participants) | Gender |
| Smith (2013) | Estimating the causal effect of beliefs on contributions in repeated public good games | Lab-based social dilemma | Correlational | Fictive money invested in a public good | Trust |
| Spadaro et al. (2023) | Gender differences in cooperation across 20 societies: a meta-analysis | Lab-based social dilemma | Meta-analysis | - Fictive money invested in a public good - Dichotomous choice between cooperation and defection (each associated with different payoff for the self and for the other participants)  - Resource taken from a common pool | Gender |
| Sturm et al. (2019) | Conditional cooperation in case of a global public good – Experimental evidence from climate change mitigation in Beijing | Real-life social dilemma | Experimental | Purchase of actions in favor of an initiative to mitigate CO2 | Trust |
| Sun et al. (2023) | Intuitive thinking impedes cooperation by decreasing cooperative expectations for pro-self but not for pro-social individuals | Lab-based commons dilemma | Experimental | Dichotomous choice between cooperation and defection (each associated with different payoff for the self and for the other participants) | Values and personality traits |
| Sussman et al. (2016) | Pro-Environmental Values Matter in Competitive but Not Cooperative Commons Dilemmas | Lab-based commons dilemma | Experimental | Resource taken from a common pool | Values and personality traits |
| Thøgersen (2008) | Social norms and cooperation in real-life social dilemmas | Real-life social dilemma | Correlational | Environmentally responsible behaviors | Social norms |
| Torsvik et al. (2011) | Anticipated discussion and cooperation in a social dilemma | Lab-based social dilemma | Experimental | Resource taken from a common pool | Communication |
| Van Dijk et al. (2015) | Promoting cooperation in social dilemmas: the use of sanctions | Lab-based social dilemma | Literature review | Actions for the collective interest | Incentives |
| Van Klingeren and Buskens (2024) | Graduated sanctioning, endogenous institutions and sustainable cooperation in common-pool resources: An experimental test | Lab-based commons dilemma | Experimental | Resource taken from a common pool | Incentives |
| Van Klingeren and De Graaf (2021) | Heterogeneity, trust and common-pool resource management | Real-life commons | Correlational | Quality of the common pool resource | Trust |
| Van Lange et al. (2013) | The psychology of social dilemmas: A review | Lab-based social dilemma | Literature review | Actions for the collective interest | Review of several factors impacting cooperation |
| Van Lange et al. (1998) | A Social Dilemma Analysis of Commuting Preferences: The Roles of Social Value Orientation and Trust | Real-life social dilemma | Experimental | Taking public transports to commute | Values and personality traits |
| Van Vugt (2009) | Averting the Tragedy of the Commons: Using Social Psychological Science to Protect the Environment | Real-life commons | Literature review | Successful common resources management | Review of several factors impacting cooperation |
| Van Vugt and Hardy (2010) | Cooperation for reputation: Wasteful contributions as costly signals in public goods | Lab-based social dilemma | Experimental | Fictive money invested in a public good | Anonymity |
| Van Vugt et al. (2007) | Gender differences in cooperation and competition: The Male-Warrior Hypothesis | Lab-based social dilemma | Experimental | Fictive money invested in a public good | Gender |
| Volk et al. (2011) | Personality, personal values and cooperation preferences in public goods games: A longitudinal study | Lab-based social dilemma | Correlational | Fictive money invested in a public good | Values and personality traits |
| Von Borgstede et al. (2018) | Social Dilemmas: Motivational, Individual, and Structural Aspects Influencing Cooperation | Real-life social dilemma | Literature review | Actions for the collective interest | Social norms |
| Wang et al. (2017) | Onymity promotes cooperation in social dilemma experiments | Lab-based social dilemma | Experimental | Dichotomous choice between cooperation and defection (each associated with different payoff for the self and for the other participants) | Anonymity |
| Waring and Bell (2013) | Ethnic dominance damages cooperation more than ethnic diversity: results from multi-ethnic field experiments in India | Real-life commons | Experimental | Fictive money invested in a public good | Social status |
| Weber and Murnighan (2008) | Suckers or saviors? Consistent contributors in social dilemmas | Lab-based social dilemma | Experimental | Dichotomous choice between cooperation and defection (each associated with different payoff for the self and for the other participants) | Social norms |
| Weimann et al. (2019) | Public good provision by large groups – the logic of collective action revisited | Lab-based social dilemma | Experimental | Fictive money invested in a public good | Anonymity |
| Wit and Wilke (1992) | The effect of social categorization on cooperation in three types of social dilemmas | Lab-based social dilemma | Experimental | Dichotomous choice between cooperation and defection (each associated with different payoff for the self and for the other participants) | Social identification |
| Wu et al. (2016) | Gossip versus punishment: The efficiency of reputation to promote and maintain cooperation | Lab-based social dilemma | Experimental | Fictive money invested in a public good | Communication |
| Xiao and Kunreuther (2016) | Punishment and Cooperation in Stochastic Social Dilemmas | Lab-based social dilemma | Experimental | Dichotomous choice between cooperation and defection (each associated with different payoff for the self and for the other participants) | Incentives |
| Yoeli et al. (2013) | Powering up with indirect reciprocity in a large-scale field experiment | Real-life commons | Experimental | Participation in a program to help prevent blackouts | Anonymity |
| Zhang (2019) | Common fate motivates cooperation: The influence of risks on contributions to public goods | Lab-based social dilemma | Experimental | Fictive money invested in a public good | Social identification |
| Zhang et al. (2023) | Does similarity trigger cooperation? Dyadic effect of similarity in social value orientation and cognitive resources on cooperation | Lab-based social dilemma | Experimental | Dichotomous choice between cooperation and defection (each associated with different payoff for the self and for the other participants) | Values and personality traits |
