# Supplementary material for: Exploring individual and organizational factors influencing cooperation in commons: a scoping review
Source: Front Psychol. 2025 Jun 3;16:1465057. doi: 10.3389/fpsyg.2025.1465057 (PMC12170531; doi:10.3389/fpsyg.2025.1465057)
Supplement: Supplementary file 3 [file Supplementary_file_3.docx]

**Appendix C**

**Summary of the main effects of each factor and of their moderators**

| Factor | Type of factor | N analyzed papers | General effect on cooperation | Moderators |
| --- | --- | --- | --- | --- |
| Gender | Individual | 13  Balliet et al. (2011a); Barrero-Amórtegui and Maldonado (2021); Colman et al. (2018); Dorrough and Glöckner (2019); Gomez-Ruiz and Sánchez-Expósito (2020); Haucap et al. (2024); Irwin et al. (2015); Peshkovskaya et al. (2017); Peshkovskaya et al. (2019); Sell and Kuipers (2009); Simpson (2003); Spadaro et al. (2023); Van Vugt et al. (2007) | Ambiguous | Relationship with other participants: - Men are more cooperative towards friends than women, while women are more cooperative towards strangers - Men cooperate more than women if they can socialize with other participants - Men improve their cooperation if a team identity is created, while this has no impact on women - In repeated interactions men, but not women, increase their cooperation over time.  Intergroup comparison: - Men cooperate more if an intergroup comparison is salient than if such a comparison is not salient. Women display the inverse pattern, cooperating less in a context of intergroup comparison. Men also display a stronger identification with their group in the intergroup comparison condition, while women’s identification do not change significatively.  Group composition: - Men are more cooperative and display higher levels of gratitude and trust in same-sex groups than women: since women are more concerned about relative payoff, defecting allows them to avoid earning less than the other players - Women cooperate more and report a stronger social connection with other members in mixed groups than men: when both genders are present, gender stereotypes activate and people tend to adapt to them. This leads women to behave in a more pro-social and altruistic way, and men to be more competitive - Groups mostly composed of women are more cooperative than those with a majority of men - This effect is also subject to moderators: for instance, a study showed that groups with a majority of women are more cooperative, but only after signing an agreement against excessive harvesting  Underlying motives: - Men cooperate less when greed incentives (i.e. temptations to free-ride) are present - Women’s principal motive in cooperative interaction is avoiding being exploited, so they cooperate less when they do not trust other participants or if they believe that others make small contributions  External impact of cooperation: - If cooperating negatively impact an external individual, women, but not men, reduce their levels of cooperation. This is explained by the fact that women display higher levels of guilt and shame aversion than men. |
| Social status | Individual | 13  Aksoy (2019); Banerjee (2024); Camera et al. (2020); Chen (2022); Drouvelis et al. (2021) ; Kingsley (2016); Malthouse et al. (2023); Peng and Fan (2023); Ramalingam and Stoddard (2024a, 2024b); Van Klingeren and De Graaf (2021); Van Lange et al. (2013); Waring and Bell (2013) | Ambiguous | Intra-class solidarity: - Low-status members discriminate against high-status members (by cooperating less with them) and by punishing them more - Low-status members punish other low-status participants significantly less for not cooperating, thus making free-riding more tempting  Group composition: - Heterogeneous groups (i.e. where different social classes are present) are less cooperative than homogeneous ones - The negative effect of heterogeneity disappears when there is an uncertainty about the outcomes - In heterogeneous groups, individuals’ beliefs about others’ contributions have a stronger impact on cooperation than in homogeneous groups - Economic and socio-cultural heterogeneity are negatively correlated with in-group trust, but there is no evidence of their negative effect on the integrity of a common-pool resource - In groups which were initially heterogeneous, the overall cooperation remains lower than in homogeneous group even when anti-inequality measures are adopted (i.e. redistributing resources between participants, or improving the endowment of low-status members). This is explained by the fact that the old high-status members don’t change their behavior, or even reduce their contributions, when the inequalities are reduced. Moreover, the old low-status members tend to imitate them by contributing less even if they have more resources. |
| Group identification | Individual | 16  Aksoy (2019); Arora et al. (2016); Böhm & Rockenbach (2013); Chen et al. (2007); De Cremer & Leonardelli (2003) ; Dorrough et al. (2015); Gomez-Ruiz and Sánchez-Expósito (2020); Jackson (2011); Jackson (2012); Kramer and Brewer (1984); Noonan et al. (2016); Nockur and Pfattheicher (2020); Puurtinen and Mappes (2009); Van Vugt (2009); Zhang (2019); Wit and Wilke (1992) | Strongly identified individuals cooperate more with the in-group than low-identified ones | Individual contributions: - Strongly identified individuals may compensate (by cooperating more) for other in-group members’ defection. However, this reduces their trust in other members.  Intergroup competition: - People cooperate more when a competition between groups is present, since it creates a context in which individuals and group interests are aligned. - In competitive contexts, individuals report more anger towards in-group free-riders and more guilt after defecting.  Superordinate identity: - Maintains the positive effects of intergroup competition, while eliminating the negative ones. - Can be primed by making salient the common characteristics of different groups (instead of their differences) or by inducing the feeling of a common fate, e.g. with a collective risk.  Group size: - Cooperation is predicted by individuals’ need to belong in big groups, while this is not the case in small groups.  Impact on other factors: - Since high-identified group members cooperate more than low-identified ones, monetary incentives are more effective at enhancing cooperation of low-identified individuals. - A previous group failure decreases cooperation between low-, but not between high-identified members. |
| Values and personality traits | Individual | 20  Balliet et al. (2009); Bilancini et al. (2022); Chen et al. (2007); De Kwaadsteniet et al. (2008); Emonds et al. (2011); Fosgaard et al. (2019); Grünhage and Reuter (2022); Hilbig et al. (2018); Kieslich and Hilbig (2014); Kocher et al. (2017); Liu and Li (2009); Lönnqvist et al. (2025); Lu et al. (2019); Probst et al. (1999); Schlösser et al. (2018); Sun et al. (2023); Sussman et al. (2016); Van Lange et al. (1998); Volk et al. (2011); Zhang et al. (2023) | Pro-social individuals cooperate more than pro-self  Pro-environmental values are positively correlated with cooperation  Left wingers are more cooperative than right wingers  Individual levels of justice sensitivity positively predict cooperation  Collectivists act for the group’s good, individualists act for their personal interest  Individuals with higher self-control cooperate more  Individuals scoring high in the Humility-Honesty dimension of the HEXACO model cooperate more than those scoring low  Individuals scoring high in the Agreeableness dimension of the Big Five model cooperate more than those scoring low | Incentives (SVO): - Since they highly value their self-interest, pro-self individuals cooperate more if economic incentives are present than if they are absent, while pro-social individuals display high levels of cooperation in both conditions. - Pro-self individuals value more the perceived efficiency of the cooperative behavior than pro-social individuals do.  Uncertainty about number of members (SVO): - Pro-social (not pro-self) individuals improve their cooperation if the number of members is not known because they seek to maximize the joint outcome and to avoid the overexploitation of the common resource.  Deliberation (SVO): - Pro-self individuals improve their cooperation if they have the possibility to deliberate about the situation, since they realize that cooperation is the best strategy to maximize the joint (and thus also personal) outcome. - If participants are forced to intuitively think (by giving them an additional cognitive task that does not allow them to focus exclusively on the cooperative interaction) the effect of SVO is amplified. - However, another result shows that SVO is not moderated by participants’ cognitive capacity during the cooperative interaction. Indeed, the effect that SVO has on cooperation is the same in a condition in which participants can deliberate and in one in which they are forced to take a quick decision.  Group composition (SVO): - In groups which are heterogeneous in terms of SVO, cooperation decreases more strongly over time than in homogeneous groups.  Other participants’ behavior (pro-environmental values): - Pro-environmental values predict cooperation only when facing non-cooperative interaction partners.  Type of cooperative interaction (political orientation): - Political orientation has a stronger effect on cooperation in situations were individuals must take resources from a common pool, than when they must contribute to a public good.  Presence of a punishment system (justice sensitivity): - The effect of justice sensitivity on cooperation is only observed in the absence of a punishment system.  Presence of a cooperative social norm (collectivism): - Collectivists people cooperate more than individualists when a cooperative social norm is present.  Effect of cooperation on personal and group outcomes (collectivism): - Individualists’ and collectivists’ motivation for cooperation differ: the former cooperate if doing so maximizes the group’s outcome, the latter cooperate if doing so maximizes their personal outcome.  Amount of others’ contributions (self-control): - The effect of self-control on cooperation is stronger the higher are others’ contributions.  Temptation to defect (Honesty-Humility): - The Honesty-Humility trait predicts cooperation when the structure of the cooperative interaction provides a strong temptation to defect. |
| Trust | Individual | 15  Balliet and Van Lange (2013a); Bechtel and Scheve (2017); Chaudhuri et al. (2002); De Cremer et al. (2001); Drouvelis et al. (2021); Franzen et al. (2019); Gätcher et al. (2004); Irlenbusch et al. (2019); Kocher et al. (2015); Lübke (2021); Oyediran et al. (2018); Rompf et al. (2017); Smith (2013); Sturm et al. (2019); Van Lange et al. (2013) | People with high levels of trust cooperate more than people with low levels | Degree of conflict between personal and collective interests: - Trust has a stronger effect in situations with a large conflict rather than a small conflict.  Uncertainty about others’ intentions: - Trust has a stronger effect if the uncertainty is high.  Anonymity: - Low truster cooperate more when their actions are accountable than in an anonymous situation, while high trusters display high levels of cooperation no matter the context.  Group composition: - In groups composed by individuals with a heterogeneous background, cooperation is more strongly impacted by people’s beliefs about others contributions than in homogeneous groups.  Perceived costs and benefits of cooperative behavior: - In people with a high institutional trust, perceived pro and cons of cooperation are less predictive of their behavior, since their levels of cooperation are already high. |
| Incentives^1^ | Organizational | 33  Almeida (2023); Ambrus and Greiner (2012); Balliet et al. (2011b); Balliet and Van Lange (2013b); Chen (2022); Chen et al. (2009); Cinyabuguma et al. (2005); Drouvelis et al. (2019); Egas and Riedl (2005); Fehr and Gätcher (2000); Grechenig et al. (2010); Herrmann et al. (2008); Hilbig et al. (2012); Irwin et al. (2014); Jacquet et al. (2011); Jin et al. (2024); Kingsley (2016); Maier-Rigaud et al. (2010); Milinski and Rockenbach (2012); Mulder et al. (2006); Nelissen and Mulder (2013); Nockur et al. (2021); Noussair et al. (2024); Peng and Fan (2023); Pfattheicher et al.(2018); Rand et al. (2009); Rockenbach and Wolff (2019); Schlösser et al. (2018); Van Dijk et al. (2015); Van Klingeren and Buskens (2024); Van Vugt (2009); Wu et al. (2016); Xiao and Kunreuther (2016) | Punishment and reward systems have a positive effect on cooperation | Cost of implementation: - Incentives are more effective if their implementation is costly rather than free.  Type of interaction: - The effect of incentives is stronger in iterated, rather than single, interactions, since in short interactions punishers are the ones with the lowest final outcome and this discourages application of the fine.  Other participants and group homogeneity: - Punishments increase cooperation only in homogeneous groups. In fact, in heterogeneous groups, low-status members tend to punish other low-status individuals less, thus encouraging free-riding. - Punishments are more effective in interactions among friends than among strangers.  Organization: - Punishments are more effective when only cooperators have the power to punish defectors, compared to a situation where everybody can distribute fines. In fact, if there are no rules about who can mete out punishment, defectors may punish cooperation as a form of retaliation. - The effect of punishments is stronger in plain situations, where the link between behavior and outcome is clear and punishments are highly effective. - The most successful groups punish with caution and not in the early stages of the interaction, and they have social norms about when and how a punishment should be applied. - A democratic punishment system (where a fine is given only if a majority agrees) is more effective than a system where everybody can decide individually about punishment: in a democratic system, punishment levels are lower, while cooperation rates and individuals’ final payoff are higher.  Accuracy of information about others’ contributions: - Less accuracy about others’ contribution (i.e. non null probability that a cooperator is seen as a defector due to inaccurate information) increases punishment levels, but without improving cooperation. This is explained by the fact that unjustly punished participants are less likely to cooperate in the following interactions.  Cultural context: - Punishment is more effective when the societal level of trust is high than when it is low, because in high-trust societies members adhere more strongly to norms of cooperation and of punishment of defectors. - Punishment does not have a positive effect on cooperation in all cultures: when the cultural norm of civic cooperation is low, the levels of antisocial punishment are higher, and consequently cooperation is lower. Type of incentives: - Cooperation drops when economic incentives are removed, but not after the removal of social ones. - The positive effect of social incentives on cooperation diminishes over time, while this is not the case for economic incentives.  Impact on other factors: - Incentives reduce participants’ intrinsic motivation to cooperate: if a punishment system is present, individual levels of justice sensitivity, or the Honesty-Humility trait (from the HEXACO personality model) impact less cooperation, because the cooperative interaction becomes strategic. - Incentives reduce participants’ trust in each other. |
| Social norms | Organizational | 12  Arora et al. (2012); Bardsley and Sausgruber (2005); Bicchieri (2002); Chaudhuri and Paichayontvijit (2017); Hassan et al. (2023); Irlenbusch et al. (2019); Lavallee et al. (2024); Rege and Telle (2004); Thøgersen (2008); Van Lange et al. (2013); Von Borgstede et al. (2018); Weber and Murnighan (2008) | Cooperative social norms increase cooperation | Anonymity: - If behaviors are observable (and thus social approval is possible) people comply more with social norms and cooperate more.  Content of the descriptive norm: - The content of the social norms defines the harvesting rate: if the norm is cooperative, individuals adapt by harvesting their fair share of the resource, while, if the norm is to over-harvest, individuals try to communicate “right” (i.e. cooperative) norm by harvesting less than their fair share. This effect is not significantly impacted by the individuals’ level of power-wealth values.  Modification of the (salience of the) social norms: - If the social norms is to defect, inducing a feeling of pride in the few cooperators, or appealing to the defectors’ values (such as religious, patriotic, or social values) can encourage cooperation. - Appealing to participants’ goodwill can be more successful than implementing a punishment system in increasing cooperation. - The presence of consistent cooperators (i.e. individuals who always cooperate) creates a more cooperative social norms. - Social norms can be shaped via communication. - Communicating others’ contribution increases cooperation. More specifically, the most effective feedback is to communicate the highest contribution of the group without specifying that it is the highest. |
| Communication | Organizational | 16  Adams et al. (2022); Balliet (2010); Baum et al. (2012); Bicchieri (2002); Feinberg et al. (2012); Feinberg et al. (2014); Ghate et al. (2013); Giardini et al. (2021); Hoenow and Pourviseh (2024); Hopthrow and Hulbert (2005); Janssen et al. (2014); Jin et al. (2024); Koessler et al. (2021a); Kumakawa (2013); Torsvik et al. (2011); Wu et al. (2016) | Having the possibility to communicate with other members increases cooperation | Group size:  - The effect of communication is stronger in large – rather than small – groups because it makes it possible to buffer the negative effects of large groups on cooperation.  Type of communication: - Oral communication has a stronger effect on cooperation than written messages because it enables non-verbal communication and facilitates the formation process of social norms. - Communication has a weaker effect if it requires a personal cost than if it is free.  Content: - Communication has a stronger effect on cooperation when it is used to raise awareness about the problem, identify possible strategies, and agree on the best strategy to apply. - Groups that use communication to foster a social identity and to promote cooperative social norms cooperate more than other groups.  Timing: - Communication enhances cooperation if individuals can exchange before or during the cooperative interaction. - Knowing that the discussion will take place after the interaction can have a negative effect on cooperation because people are forced to reflect about the situation and therefore take more rational decisions.  Context: - In a real-life context, where participants already know each other, cooperation is high even in absence of communication. Communication has, however, a positive effect on cooperation, because it increases the homogenization of the cooperative behavior and the equitable distribution of payoffs. |
| Anonymity | Organizational | 17  Butz and Harbring (2021); Christens et al. (2019); De Cremer et al. (2001); Hil and Gurven (2005); Jiang et al. (2021); Jin et al. (2024); Koessler et al. (2021b); Ma et al. (2024); Mosler (1993); Przepiorka and Diekmann (2020); Rege and Telle (2004); Romano et al. (2016); Van Vugt and Hardy (2010); Von Borgstede et al. (2018); Wang et al. (2017); Weimann et al. (2019); Yoelia et al. (2013) | People cooperate more in non-anonymous situations than in anonymous ones | Personal costs and benefits: - The difference in cooperation between anonymous and non-anonymous situations is stronger if cooperation is costly and does not bring any personal benefit because it shows that there are no personal interests that could explain cooperation. People are therefore perceived as altruistic, which is a socially valued characteristic.  Individual level of image concern: - The positive effect of making a situation non-anonymous is stronger for individuals with high image concern, who are concerned about what others may think of them and of their actions.  Trust: - Low truster cooperate more when their actions are accountable than in an anonymous situation, while high trusters display high levels of cooperation no matter the context. |

^1^ “Incentives” is used when the effect applies to both reward and punishment. Otherwise, the specific terms are used.
Note: the general effect on cooperation is reported as “Ambiguous” when the analyzed papers did not allow to draw a clear conclusion about how these factors affect cooperation. Otherwise, the main effect we identified is reported.
