# Supplementary material for: Exploring individual and organizational factors influencing cooperation in commons: a scoping review
Source: Front Psychol. 2025 Jun 3;16:1465057. doi: 10.3389/fpsyg.2025.1465057 (PMC12170531; doi:10.3389/fpsyg.2025.1465057)
Supplement: Supplementary file 4 [file Supplementary_file_4.docx]

**Appendix D**

**Quality assessment of the 105 papers included in the scoping review**

| Author(s) and date | Title | Journal | Peer-reviewed journal^1^ | Cite Score 2023^2^ | H Index 2023^3^ |
| --- | --- | --- | --- | --- | --- |
| Adams et al. (2022) | Verbal interaction in a social dilemma | Rationality and Society | yes | 1.5 | 52 |
| Aksoy (2019) | Crosscutting circles in a social dilemma: Effects of social identity and inequality on cooperation | Social Science Research | yes | 4.3 | 112 |
| Almeida (2023) | Punishment credibility and cooperation in public good games | Journal of Behavioral and Experimental Economics | yes | 2.6 | 77 |
| Ambrus and Greiner (2012) | Imperfect public monitoring with costly punishment: An experimental study | American Economic Review | yes | 18.6 | 359 |
| Arora et al. (2016) | Acting for the Greater Good: Identification with Group Determines Choices in Sequential Contribution Dilemmas: Group Identity and Sequential Social Dilemmas | Journal of Behavioral Decision Making | yes | 4.4 | 92 |
| Arora et al. (2012) | To cooperate or not to cooperate: Using new methodologies and frameworks to understand how affiliation influences cooperation in the present and future | Journal of Economic Psychology | yes | 5.2 | 118 |
| Balliet (2010) | Communication and cooperation in social dilemmas: A meta-analytic review | Journal of Conflict Resolution | yes | 5.3 | 124 |
| Balliet and Van Lange (2013) | Trust, Punishment, and Cooperation Across 18 Societies: A Meta-Analysis | Perspectives on Psychological Science | yes | 22.7 | 181 |
| Balliet and Van Lange (2013) | Trust, conflict, and cooperation: A meta-analysis | Psychological Bulletin | yes | 33.6 | 359 |
| Balliet et al. (2011) | Sex differences in cooperation: A meta-analytic review of social dilemmas | Psychological Bulletin | yes | 33.6 | 359 |
| Balliet et al. (2011) | Reward, punishment, and cooperation: A meta-analysis | Psychological Bulletin | yes | 33.6 | 359 |
| Balliet et al. (2009) | Social Value Orientation and cooperation in social dilemmas: A meta-analysis | Group Processes & Intergroup Relations | yes | 8.5 | 93 |
| Banerjee (2024) | The effect of heterogeneity and risk on co-operation: experimental evidence | Journal of Behavioral and Experimental Economics | yes | 2.6 | 77 |
| Bardsley and Sausgruber (2005) | Conformity and reciprocity in public good provision | Journal of Economic Psychology | yes | 5.2 | 118 |
| Barrero-Amórtegui and Maldonado (2021) | Gender composition of management groups in a conservation agreement framework: Experimental evidence for mangrove use in the Colombian Pacific | World Development | yes | 12.7 | 219 |
| Baum et al. (2012) | Cooperation due to cultural norms, not individual reputation | Behavioural Processes | yes | 2.7 | 91 |
| Bechtel and Scheve (2017) | Who Cooperates? Reciprocity and the Causal Effect of Expected Cooperation in Representative Samples | Journal of Experimental Political Science | yes | 5.1 | 23 |
| Bicchieri (2002) | Covenants without swords: group identity, norms, and communication in social dilemmas | Rationality and Society | yes | 1.5 | 52 |
| Bilancini et al. (2022) | Social value orientation and conditional cooperation in the online one-shot public goods game | Journal of Economic Behavior & Organization | yes | 3.8 | 136 |
| Böhm and Rockenbach (2013) | The Inter-Group Comparison – Intra-Group Cooperation Hypothesis: Comparisons between Groups Increase Efficiency in Public Goods Provision | PLoS ONE | yes | 6.2 | 435 |
| Butz and Harbring (2021) | The effect of disclosing identities in a socially incentivized public good game | Games | yes | 1.6 | 22 |
| Camera et al. (2020) | Do economic inequalities affect long-run cooperation and prosperity? | Experimental Economics | yes | 4.1 | 65 |
| Chaudhuri and Paichayontvijit (2017) | On the long-run efficacy of punishments and recommendations in a laboratory public goods game | Scientific Reports | yes | 7.5 | 315 |
| Chaudhuri et al. (2002) | Cooperation in social dilemmas, trust and reciprocity | Journal of Economic Psychology | yes | 5.2 | 118 |
| Chen et al. (2009) | Unintended Consequences of Cooperation Inducing and Maintaining Mechanisms in Public Goods Dilemmas: Sanctions and Moral Appeals | Group Processes & Intergroup Relations | yes | 8.5 | 93 |
| Chen (2022) | Carrots and sticks: new evidence in public goods games with heterogeneous groups | International Journal of Intercultural Relations | yes | 4.3 | 102 |
| Chen et al. (2007) | When does group norm or group identity predict cooperation in a public goods dilemma? The moderating effects of idiocentrism and allocentrism | Journal of Economic Interaction and Coordination | no | 2.2 | 27 |
| Christens et al. (2019) | Identification of individuals and groups in a public goods experiment | Journal of Behavioral and Experimental Economics | yes | 2.6 | 77 |
| Cinyabuguma et al. (2005) | Cooperation under the threat of expulsion in a public goods experiment | Journal of Public Economics | yes | 14.1 | 174 |
| Colman et al. (2018) | Persistent cooperation and gender differences in repeated Prisoner’s Dilemma games: Some things never change | Acta Psychologica | yes | 3 | 112 |
| De Cremer and Leonardelli (2003) | Cooperation in social dilemmas and the need to belong: The moderating effect of group size | Group Dynamics: Theory, Research, and Practice | yes | 6.5 | 75 |
| De Cremer et al. (2001) | ‘The less I trust, the less I contribute (or not)?’ The effects of trust, accountability and self‐monitoring in social dilemmas | European Journal of Social Psychology | Yes | 6.1 | 134 |
| De Kwaadsteniet et al. (2008) | ‘How many of us are there?’: Group size uncertainty and social value orientations in common resource dilemmas | Group Processes & Intergroup Relations | yes | 8.5 | 93 |
| Dorrough and Glöckner (2019) | A cross-national analysis of sex differences in prisoner’s dilemma games | British Journal of Social Psychology | yes | 9.5 | 114 |
| Dorrough et al. (2015) | The development of ingroup favoritism in repeated social dilemmas | Frontiers in Psychology | yes | 5.3 | 184 |
| Drouvelis et al. (2021) | Cooperation in a fragmented society: Experimental evidence on Syrian refugees and natives in Lebanon | Journal of Economic Behavior & Organization | yes | 3.8 | 136 |
| Egas and Riedl (2005) | The economics of altruistic punishment and the maintenance of cooperation | Proceedings of the Royal Society B: Biological Sciences | yes | 7.9 | 289 |
| Emonds et al. (2011) | Comparing the neural basis of decision making in social dilemmas of people with different social value orientations, a fMRI study | Journal of Neuroscience, Psychology, and Economics | yes | 1.5 | 30 |
| Fehr and Gätcher (2000) | Cooperation and punishment in public goods experiments | American Economic Review | yes | 18.6 | 359 |
| Feinberg et al. (2014) | Gossip and ostracism promote cooperation in groups | Journal of Personality and Social Psychology | yes | 12.7 | 434 |
| Feinberg et al. (2012) | The virtues of gossip: Reputational information sharing as prosocial behavior | Psychological Science | yes | 13.3 | 316 |
| Fosgaard et al. (2019) | Cooperation, framing, and political attitudes | Journal of Economic Behavior & Organization | yes | 3.8 | 136 |
| Franzen et al. (2019) | Governing the commons: why self-administered farm outlets flourish in Switzerland | International Journal of the Commons | yes | 3.7 | 36 |
| Gätcher et al. (2004) | Trust, voluntary cooperation, and socio-economic background: survey and experimental evidence | Journal of Economic Behavior & Organization | yes | 3.8 | 136 |
| Ghate et al. (2013) | Cultural norms, cooperation, and communication: Taking experiments to the field in indigenous communities | International Journal of the Commons | yes | 3.7 | 36 |
| Giardini et al. (2021) | Gossip and competitive altruism support cooperation in a Public Good game | Philosophical Transactions of the Royal Society B: Biological Sciences | yes | 11.8 | 319 |
| Gomez-Ruiz and Sánchez-Expósito (2020) | The impact of team identity and gender on free-riding responses to fear and cooperation sustainability | Sustainability | yes | N/A | 169 |
| Grechenig et al. (2010) | Punishment despite reasonable doubt—A public goods experiment with sanctions under uncertainty | Journal of Empirical Legal Studies | yes | 2.3 | 36 |
| Grünhage and Reuter (2022) | Political orientation is associated with behavior in public-goods- and trust-games | Political Behavior | yes | 8.4 | 95 |
| Hassan et al. (2023) | Incentivizing cooperation against a norm of defection: experimental evidence from Egypt | Journal of Behavioral and Experimental Economics | yes | 2.6 | 77 |
| Haucap et al. (2024) | Gender and cooperation in the presence of negative externalities | Games and Economic Behavior | yes | 1.9 | 106 |
| Herrmann et al. (2008) | Antisocial Punishment Across Societies | Science | yes | 61.1 | 1336 |
| Hilbig et al. (2012) | Personality, Punishment and Public Goods: Strategic Shifts Towards Cooperation as a Matter of Dispositional Honesty–Humility | European Journal of Personality | yes | 11.9 | 103 |
| Hilbig et al. (2018) | Lead us (not) into temptation: Testing the motivational mechanisms linking honesty–humility to cooperation | European Journal of Personality | yes | 11.9 | 103 |
| Hill and Gurven (2004) | 13 Economic Experiments to Examine Fairness and Cooperation among the Ache Indians of Paraguay | [book chapter] | N/A | N/A | N/A |
| Hoenow and Pourviseh (2024) | Intragroup communication in social dilemmas: An artefactual public good field experiment in small-scale communities | Judgment and Decision Making | yes | 4.4 | 73 |
| Hopthrow and Hulbert (2005) | The effect of group decision making on cooperation in social dilemmas | Group Processes & Intergroup Relations | yes | 8.5 | 93 |
| Irlenbusch et al. (2019) | Designing feedback in voluntary contribution games: the role of transparency | Experimental Economics | yes | 4.1 | 65 |
| Irwin et al. (2015) | Gender, trust and cooperation in environmental social dilemmas | Social Science Research | yes | 4.3 | 112 |
| Irwin et al. (2014) | The detrimental effects of sanctions on intragroup trust: Comparing punishments and rewards | Social Psychology Quarterly | yes | 4.1 | 102 |
| Jackson (2011) | Intragroup cooperation as a function of group performance and group identity | Group Dynamics: Theory, Research, and Practice | yes | 6.5 | 75 |
| Jackson (2012) | Reactions to a social dilemma as a function of intragroup interactions and group performance | Group Processes & Intergroup Relations | yes | 8.5 | 93 |
| Jacquet et al. (2011) | Shame and honour drive cooperation | Biology Letters | yes | 5.5 | 134 |
| Janssen et al. (2014) | The effect of constrained communication and limited information in governing a common resource | International Journal of the Commons | yes | 3.7 | 36 |
| Jiang et al. (2021) | Reducing the bystander effect via decreasing group size to solve the collective-risk social dilemma | Applied Mathematics and Computation | yes | 7.9 | 174 |
| Jin et al. (2024) | Institutions and cooperation: A meta-analysis of structural features in social dilemmas | Journal of Personality and Social Psychology | yes | 12.7 | 434 |
| Kieslich and Hilbig (2014) | Cognitive conflict in social dilemmas: An analysis of response dynamics | Judgment and Decision Making | yes | 4.4 | 73 |
| Kingsley (2016) | Endowment heterogeneity and peer punishment in a public good experiment: Cooperation and normative conflict | Journal of Behavioral and Experimental Economics | yes | 2.6 | 77 |
| Kocher et al. (2015) | The role of beliefs, trust, and risk in contributions to a public good | Journal of Economic Psychology | yes | 5.2 | 118 |
| Kocher et al. (2017) | Strong, bold, and kind: self-control and cooperation in social dilemmas | Experimental Economics | yes | 4.1 | 65 |
| Koessler, Ortiz-Riomalo, et al. (2021) | Structuring communication effectively—The causal effects of communication elements on cooperation in social dilemmas | Environmental and Resource Economics | yes | 10.2 | 112 |
| Koessler, Page, et al. (2021) | Public cooperation statements | Journal of Economic Interaction and Coordination | no | 2.2 | 27 |
| Kramer and Brewer (1984) | Effects of group identity on resource use in a simulated commons dilemma | Journal of Personality and Social Psychology | yes | 12.7 | 434 |
| Kumakawa (2013) | Evaluating others’ behaviour: a public-good experiment with ex-post communication | Applied Economics Letters | yes | 2.9 | 63 |
| Lavallee et al. (2024) | A conservation orientation in commons dilemmas | Journal of Environmental Psychology | yes | 10.6 | 181 |
| Liu and Li (2009b) | Contextualized self: When the self runs into social dilemmas | International Journal of Psychology | yes | 6.4 | 78 |
| Lönnqvist et al. (2025) | Ideological constraint and behavioral consistency—A person-centered approach to political attitudes and Public Goods Games behavior | Frontiers in Social Psychology | yes | NA | NA |
| Lu et al. (2019) | True versus strategic fairness in a common resource dilemma: Evidence from the dual‐process perspective | Journal of Behavioral Decision Making | yes | 4.4 | 92 |
| Lübke (2021) | The climate change dilemma: How cooperation beliefs influence energy conservation behavior | Sustainability | yes | N/A | 169 |
| Ma et al. (2024) | High level of self-disclosure on SNSS facilitates cooperation: A serial mediation model of psychological distance and trust | Computers in Human Behavior | yes | 7.8 | 251 |
| Maier-Rigaud et al. (2010) | Ostracism and the provision of a public good: experimental evidence | Journal of Economic Behavior & Organization | yes | 3.8 | 136 |
| Malthouse et al. (2023) | When fairness is not enough: The disproportionate contributions of the poor in a collective action problem | Journal of Experimental Psychology: General | yes | 6.2 | 186 |
| Milinski and Rockenbach (2012) | On the interaction of the stick and the carrot in social dilemmas | Journal of Theoretical Biology | yes | 4.2 | 173 |
| Mosler (1993) | Self-dissemination of environmentally-responsible behavior: The influence of trust in a commons dilemma game | Journal of Environmental Psychology | yes | 10.6 | 181 |
| Mulder et al. (2006) | Undermining trust and cooperation: The paradox of sanctioning systems in social dilemmas | Journal of Experimental Social Psychology | yes | 6.3 | 171 |
| Nelissen and Mulder (2013) | What makes a sanction “stick”? The effects of financial and social sanctions on norm compliance | Social Influence | yes | 1.5 | 36 |
| Nockur and Pfattheicher (2020) | Fostering sustainable behavior through group competition | Journal of Environmental Psychology | yes | 10.6 | 181 |
| Nockur et al. (2021) | Different punishment systems in a public goods game with asymmetric endowments | Journal of Experimental Social Psychology | yes | 6.3 | 171 |
| Noonan et al. (2016) | Characteristics of voluntary behavior in the neighborhood commons: The case of dog parks | Nonprofit and Voluntary Sector Quarterly | yes | 5.3 | 101 |
| Noussair et al. (2024) | The role of emotions in public goods games with and without punishment opportunities | Journal of Economic Behavior & Organization | yes | 3.8 | 136 |
| Oyediran et al. (2018) | Cooperation and optimism in a social dilemma | Bulletin of Economic Research | yes | 1.4 | 35 |
| Peng and Fan (2023) | Incomplete punishment networks, heterogeneity, and cooperation in public good experiments | Journal of Behavioral and Experimental Economics | yes | 2.6 | 77 |
| Peshkovskaya et al. (2019) | Gender effects and cooperation in collective action: A laboratory experiment | CEUR Workshop Proceeding | no | 1.1 | 66 |
| Peshkovskaya et al. (2017) | Do women socialize better? Evidence from a study on sociality effects on gender differences in cooperative behavior’ | Rationality and Society | yes | 1.5 | 52 |
| Pfattheicher et al. (2018) | The advantage of democratic peer punishment in sustaining cooperation within groups | Journal of Behavioral Decision Making | yes | 4.4 | 92 |
| Probst et al. (1999) | Cultural values in intergroup and single-group social dilemmas | Organizational Behavior and Human Decision Processes | yes | 8.9 | 177 |
| Przepiorka and Diekmann (2020) | Binding contracts, non-binding promises and social feedback in the intertemporal common-pool resource game | Games | yes | 1.6 | 22 |
| Puurtinen and Mappes (2009) | Between-group competition and human cooperation | Proceedings of the Royal Society B: Biological Sciences | yes | 7.9 | 289 |
| Ramalingam and Stoddard (2024a) | Does reducing inequality increase cooperation? | Journal of Economic Behavior & Organization | yes | 3.8 | 136 |
| Ramalingam and Stoddard (2024b) | Inequality reduction and cooperation: Injection of additional resources | Economics Letters | yes | 3.2 | 125 |
| Rand et al. (2009) | Positive Interactions Promote Public Cooperation | Science | yes | 61.1 | 1336 |
| Rege and Telle (2004) | The impact of social approval and framing on cooperation in public good situations | Journal of Public Economics | yes | 14.1 | 174 |
| Rockenbach and Wolff (2019) | The dose does it: Punishment and cooperation in dynamic public-good games | Review of Behavioral Economics | yes | 2.1 | 5 |
| Romano et al. (2016) | On the role of group size in social dilemmas | Psicologia sociale | yes | 2.1 | 12 |
| Rompf et al. (2017) | Institutional trust and the provision of public goods: When do individual costs matter? The case of recycling | Rationality and Society | yes | 1.5 | 52 |
| Schlösser et al. (2018) | Justice sensitivity and cooperation dynamics in repeated public good games | Social Justice Research | yes | 3.3 | 63 |
| Sell and Kuipers (2009) | A structural social psychological view of gender differences in cooperation | Sex Roles | yes | 7.2 | 145 |
| Simpson (2003) | Sex, fear, and greed: A social dilemma analysis of gender and cooperation | Social Forces | yes | 6.3 | 151 |
| Smith (2013) | Estimating the causal effect of beliefs on contributions in repeated public good games | Experimental Economics | yes | 4.1 | 65 |
| Spadaro et al. (2023) | Gender differences in cooperation across 20 societies: a meta-analysis | Philosophical Transactions of the Royal Society B: Biological Sciences | yes | 11.8 | 319 |
| Sturm et al. (2019) | Conditional cooperation in case of a global public good – Experimental evidence from climate change mitigation in Beijing | China Economic Review | yes | 10.6 | 101 |
| Sun et al. (2023) | Intuitive thinking impedes cooperation by decreasing cooperative expectations for pro-self but not for pro-social individuals | The Journal of Social Psychology | yes | 4.4 | 91 |
| Sussman et al. (2016) | Pro-Environmental Values matter in competitive but not cooperative commons dilemmas | The Journal of Social Psychology | yes | 4.4 | 91 |
| Thøgersen (2008) | Social norms and cooperation in real-life social dilemmas | Journal of Economic Psychology | yes | 5.2 | 118 |
| Torsvik et al. (2011) | Anticipated discussion and cooperation in a social dilemma | Rationality and Society | yes | 1.5 | 52 |
| Van Dijk et al. (2015) | Promoting cooperation in social dilemmas: the use of sanctions | Current Opinion in Psychology | yes | 12.1 | 89 |
| Van Klingeren and Buskens (2024) | Graduated sanctioning, endogenous institutions and sustainable cooperation in common-pool resources: An experimental test | Rationality and Society |  | 1.5 | 52 |
| Van Klingeren and De Graaf (2021) | Heterogeneity, trust and common-pool resource management | Journal of Environmental Studies and Sciences | yes | 3.6 | 37 |
| Van Lange et al. (2013) | The psychology of social dilemmas: A review | Journal of Applied Social Psychology | yes | 4.3 | 134 |
| Van Lange et al. (1998) | A social dilemma analysis of commuting preferences: The roles of Social Value Orientation and trust | Organizational Behavior and Human Decision Processes | yes | 8.9 | 177 |
| Van Vugt et al. (2007) | Gender Differences in Cooperation and Competition: The Male-Warrior Hypothesis | Psychological Science | yes | 13.3 | 316 |
| Van Vugt (2009) | Averting the Tragedy of the Commons: Using social psychological science to protect the environment | Current Directions in Psychological Science | yes | 13 | 207 |
| Van Vugt and Hardy (2010) | Cooperation for reputation: Wasteful contributions as costly signals in public goods | Group Processes & Intergroup Relations | yes | 8.5 | 93 |
| Volk et al. (2011) | Personality, personal values and cooperation preferences in public goods games: A longitudinal study | Personality and Individual Differences | yes | 8.5 | 205 |
| Von Borgstede et al. (2018) | Social dilemmas: motivational, individual, and structural aspects influencing cooperation | [book chapter] | N/A | N/A | N/A |
| Wang et al. (2017) | Onymity promotes cooperation in social dilemma experiments | Science Advances | yes | 21.4 | 252 |
| Waring and Bell (2013) | Ethnic dominance damages cooperation more than ethnic diversity: results from multi-ethnic field experiments in India | Evolution and Human Behavior | yes | 8.3 | 130 |
| Weber and Murnighan (2008) | Suckers or saviors? Consistent contributors in social dilemmas | Journal of Personality and Social Psychology | yes | 12.7 | 434 |
| Weimann et al. (2019) | Public good provision by large groups – the logic of collective action revisited | European Economic Review | yes | 4.7 | 147 |
| Wit and Wilke (1992) | The effect of social categorization on cooperation in three types of social dilemmas | Journal of Economic Psychology | yes | 5.2 | 118 |
| Wu et al. (2016) | Gossip Versus Punishment: The Efficiency of Reputation to Promote and Maintain Cooperation | Scientific Reports | yes | 7.5 | 315 |
| Xiao and Kunreuther (2016) | Punishment and Cooperation in Stochastic Social Dilemmas | Journal of Conflict Resolution | yes | 5.3 | 124 |
| Yoeli et al. (2013) | Powering up with indirect reciprocity in a large-scale field experiment | Proceedings of the National Academy of Sciences | yes | 19 | 869 |
| Zhang (2019) | Common fate motivates cooperation: The influence of risks on contributions to public goods | Journal of Economic Psychology | yes | 5.2 | 118 |
| Zhang et al. (2023) | Does similarity trigger cooperation? Dyadic effect of similarity in social value orientation and cognitive resources on cooperation | Current Psychology | yes | 4.6 | 59 |

^1^ The information concerning the peer-review status of the journals have been retrieved on: <http://ulrichsweb.serialssolutions.com/>

^2^ The information concerning the Cite Score for the year 2023 have been retrieved on: <https://www.scopus.com/sources.uri>. The color represents the quartile of the journal in its discipline, based on the highest percentile provided by the above-mentioned website. Green = 1^st^ quartile, yellow = 2^nd^ quartile, orange = 3^rd^ quartile, red = 4^th^ quartile.

^3^ The information concerning the H Index 2023 have been retrieved on: <https://www.scimagojr.com/>. The color represents the quartile of the journal in its discipline. If the journal was classed in more than one discipline, the median of the different quartiles was taken. Green = 1^st^ quartile, yellow = 2^nd^ quartile, orange = 3^rd^ quartile, red = 4^th^ quartile.
